# Recent advances in near-infrared-II hollow nanoplatforms for photothermal-based cancer treatment

**DOI:** 10.1186/s40824-022-00308-z

**Published:** 2022-11-08

**Authors:** Li Zhang, Gerile Oudeng, Feiqiu Wen, Guangfu Liao

**Affiliations:** 1 School of Science, Harbin Institute of Technology (Shenzhen), 518055 Shenzhen, China; 2 Department of Hematology and Oncology, Shenzhen Children’s Hospital, Futian, Guangdong Shenzhen, PR China; 3 College of Material Engineering, Fujian Agriculture and Forestry University, 350002 Fuzhou, PR China

**Keywords:** Near-infrared-II, Photothermal therapy, Hollow nanoplatforms, Synergistic cancer treatment, Nanotheranostics

## Abstract

Near-infrared-II (NIR-II, 1000–1700 nm) light-triggered photothermal therapy (PTT) has been regarded as a promising candidate for cancer treatment, but PTT alone often fails to achieve satisfactory curative outcomes. Hollow nanoplatforms prove to be attractive in the biomedical field owing to the merits including good biocompatibility, intrinsic physical-chemical nature and unique hollow structures, etc. On one hand, hollow nanoplatforms themselves can be NIR-II photothermal agents (PTAs), the cavities of which are able to carry diverse therapeutic units to realize multi-modal therapies. On the other hand, NIR-II PTAs are capable of decorating on the surface to combine with the functions of components encapsulated inside the hollow nanoplatforms for synergistic cancer treatment. Notably, PTAs generally can serve as good photoacoustic imaging (PAI) contrast agents (CAs), which means such kind of hollow nanoplatforms are also expected to be multifunctional all-in-one nanotheranostics. In this review, the recent advances of NIR-II hollow nanoplatforms for single-modal PTT, dual-modal PTT/photodynamic therapy (PDT), PTT/chemotherapy, PTT/catalytic therapy and PTT/gas therapy as well as multi-modal PTT/chemodynamic therapy (CDT)/chemotherapy, PTT/chemo/gene therapy and PTT/PDT/CDT/starvation therapy (ST)/immunotherapy are summarized for the first time. Before these, the typical synthetic strategies for hollow structures are presented, and lastly, potential challenges and perspectives related to these novel paradigms for future research and clinical translation are discussed.

**Graphical Abstract:**

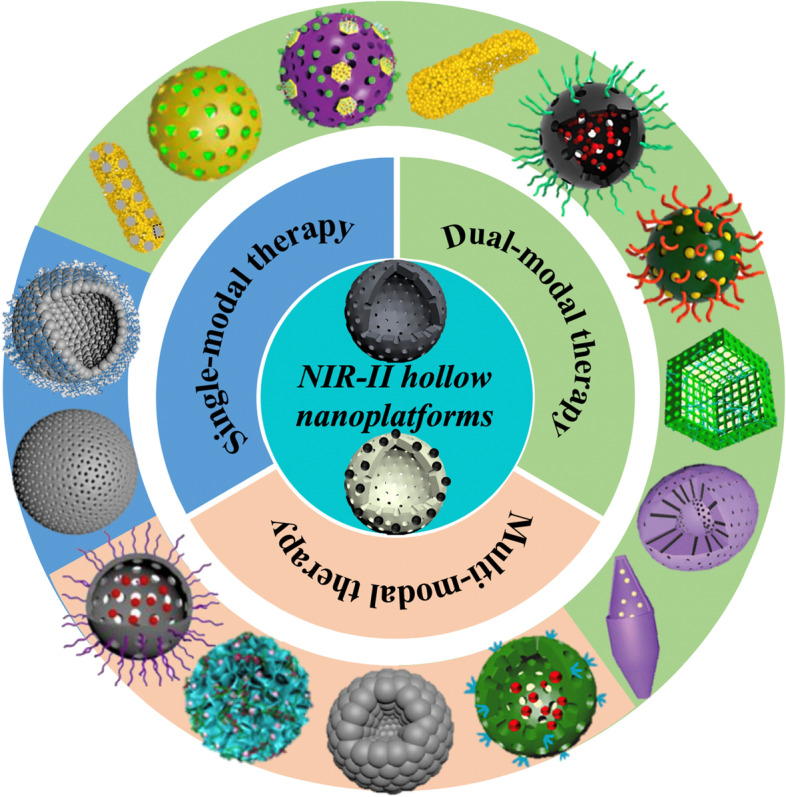

## Introduction

Cancer is known as the major public health issue globally and severely threatens human life with extremely high mortality rate [1–4]. Up to now, surgery, chemotherapy and radiotherapy have been widely applied in treating diverse tumors, but the curative outcomes are not that satisfied [5, 6]. For example, surgical resection is not able to realize complete removal of tumors because it is difficult to accurately distinguish the edge of tumorous tissues from the ambient tissues [7]. Moreover, these traditional therapeutic modalities tend to cause appendant damage to the normal tissues as the administrated small molecule drugs often suffer from non-specific biodistribution [8, 9]. The limited blood circulation time also results in poor tumor accumulation while high dose treatment regimens leads to systemic toxicities [10, 11]. Furthermore, the multidrug resistance and metastasis of tumors have always seriously restricted the therapeutic efficacy [12]. Therefore, the development of alternative treatment paradigms with superior merits is in urgent need [13–15].

Photothermal therapy (PTT) has received increasing attention in the past decade, which utilizes various photothermal agents (PTAs) to absorb near-infrared (NIR, 700–1700 nm) light and convert the light energy into hyperthermia for tumor ablation [16–18]. In contrast to the conventional treatment techniques, PTT is non-invasive and spatiotemporally controllable with higher therapeutic efficacy and low healthy-tissue damage, these advantages make PTT attractive and promising candidate for use in anti-cancer therapy [19, 20]. In short, PTAs exhibiting passive or active targeting capacities are capable of selectively accumulating in the tumor and then conducting accurate PTT with the help of localized NIR laser, since water, blood and other tissue components in body show insignificant NIR absorption [21, 22]. As we know, PTAs can be classified into two main categories, i.e. organic and inorganic nanomaterials. So far, small molecule dyes [23–25] and conjugated polymers [26–28] as the representatives of organic PTAs have been extensively explored for PTT. On the other hand, noble metal nanoparticles (NPs) [29, 30], metal chalcogenide NPs [31, 32] and 2D nanomaterials [33, 34] are popular inorganic PTAs. Each kind of PTAs possesses its own pros and pons, both organic and inorganic PTAs are very attractive in cancer PTT. In general, organic PTAs display higher absorption coefficient and biocompatibility but worse water solubility and photostability than inorganic PTAs [18, 22, 35]. Besides, the particle size and morphology of inorganic PTAs are more readily tuned, and their surfaces also tend to be much easier to modify and functionalize [36, 37].

Due to the deeper penetration depth, lower light absorption and scattering in tissues as well as higher maximum permissible exposure (MPE) intensity (1 W cm^− 2^ for 1064 nm, 0.72 W cm^− 2^ for 980 nm and 0.33 W cm^− 2^ for 808 nm), NIR-II (1000–1700 nm) PTT appears to be more superior than NIR-I (700–1000 nm) PTT, and has become the research hotpot in recent years [38–40]. For example, a comparative in vivo study has been conducted by Zhou et al. [41] using hyperbranched gold plasmonic blackbodies (AuPBs). The as-prepared AuPB displayed a broadband absorption ranging from 400 to 1350 nm and possessed a superior photothermal conversion efficiency (PCE) over 80% at 1064 nm. Utilizing a 5 mm chicken tissue to cover tumors to simulate practical situation of treating buried tumors, the temperature of the covered tumor increased to higher than 50 °C within 3 min irradiation and successfully inhibited the tumor growth. However, the covered tumors treated with PTT at 808 nm reached the maximum temperature lower than the apoptotic threshold temperature (43 °C), thus showing a negligible inhibitory effect. Typically, NIR-II PTAs should exhibit good biocompatibility, low toxicity, strong photostability, NIR-II light absorption and desirable PCE [42]. Current researches demonstrate that size control and surface engineering can be used to regulate their cytotoxicity. Such as, various polymer biomolecules modified the surface of PTAs to prolong blood circulation time, satisfied tumor accumulation, easily excrete and achieve better biocompatibility [37]. Of note, optical absorption and photothermal conversion ability are the key parameters for ideal NIR-II PTAs. As for inorganic NIR-II PTAs, broadening NIR-II absorption is most commonly adopted to improve the photothermal effects. More specifically, inorganic materials with enough low energy charge carriers and localized surface plasmonic resonance (LSPR) effect are in favor of NIR-II PTAs. For example, carbon-based nanomaterials possessed delocalized π electrons can interact with NIR-II photons to enable absorption [43]. Transition metals have partially filled d sub-shells that are likely to have low-energy electrons for NIR-II absorption [40]. The collective resonant oscillation of conduction electrons induced LSPR effect of noble metals results in an easily tuned and broadened absorption [44]. Besides, special structure design, SPR effect, surfactant modification and elements doping are able to enhance the NIR-II harvesting for enhanced PTT [45]. Additionally, the photothermal performance seriously dependent on the transformation of light to heat. Doping different elements in the other PTAs will generate carrier traps or “hot centers” to boost the photothermal conversion, and adjusting the host matrix or components of different inorganic elements can also lead to a higher PCE [46, 47]. Meanwhile, precisely diagnosing tumor and monitoring the treatment process by diverse molecular imaging techniques are of significant importance [48–51]. Among the clinical diagnostic approaches, magnetic resonance imaging (MRI), computed tomography (CT) imaging and fluorescence imaging (FLI), etc. are extensively exerted with the assistance of contrast agents (CAs) [52–54]. However, several drawbacks still exist to restrict their further applications. For example, MRI suffers from low sensitivity and relatively long acquisition time; CT imaging is not able to achieve sufficient functional information and soft tissue contrast; FLI is often accompanied by poor spatial resolution and tissue penetration [55–58]. Interestingly, PTAs are intrinsic photoacoustic imaging (PAI) CAs, that is to say, PTAs themselves are able to be all-in-one theranostic nanoplatforms [18, 38]. It is noticeable that PAI as a hybrid imaging method integrates the merits of both optical and ultrasound imaging, providing non-destructive, high-spatial resolution and deep-tissue images for cancer diagnosis [59–61].

With the ever-increasing requirement of modern medicine, single-modal imaging or treatment cannot obtain sufficient diagnostic information and satisfied curative outcomes [62–65]. To address these issues, researchers have shifted their focuses to developing multifunctional nanotheranostics [66–68]. A promising way is to incorporate these diagnostic and therapeutic functions into one single nanoplatform especially hollow nanoplatform [69–71]. Hollow nanoplatforms are accompanied by mesoporous pore structures, thus it is not uncommon to see small molecule drugs encapsulated into the cavities [72, 73]. Such novel nanoplatforms are able to serve as smart drug delivery systems (DDSs) to enhance the therapeutic outcomes of conventional chemotherapy and radiotherapy with reduced side effects [74, 75]. Compared with the mesoporous nanocarriers, hollow nanoplatforms are more popular owing to the superior drug loading capabilities. For example, only 5.6% [76] and 9.09% [77] of doxorubicin (DOX) could be encapsulated into traditional mesoporous silica NPs, while the hollow mesoporous silica NPs showed a much higher DOX loading efficacy of 42.9% [78]. In general, the synthetic strategies for hollow nanoplatforms are divided into sacrificial-template-based method and self-templating method, the details of which will be discussed in the next section. Notably, the hollow nanoplatforms alone can be good PTAs or serve as the substrates to support the deposition of PTAs [72, 79]. With other functional components combined, the hollow structured nanoplatforms are expected to allow imaging-guided PTT-based treatments. For example, Zheng et al. [80] loaded manganese carbonyl (MnCO) into the cavity of hollow mesoporous copper sulphide (CuS) NPs for MRI-guided combined PTT/gas therapy. Additionally, Wang et al. [69] reported a multifunctional macrophage-mediated nanotheranostics (denoted as MFe_3_O_4_-Cy5.5) based on fluorescent probe Cy5.5-conjugated Fe_3_O_4_ NPs, which could realize multi-modal diagnose (PAI/MRI/NIR FLI), precise imaging-guided surgery and effective PTT of gliomas. By reasonable and optimal design, the hollow NIR-I nanoplatforms can also be extended to NIR-II nanosystems for synergistic diagnostic and therapeutic efficacy with enormous advantages [81, 82].

**Scheme 1:**
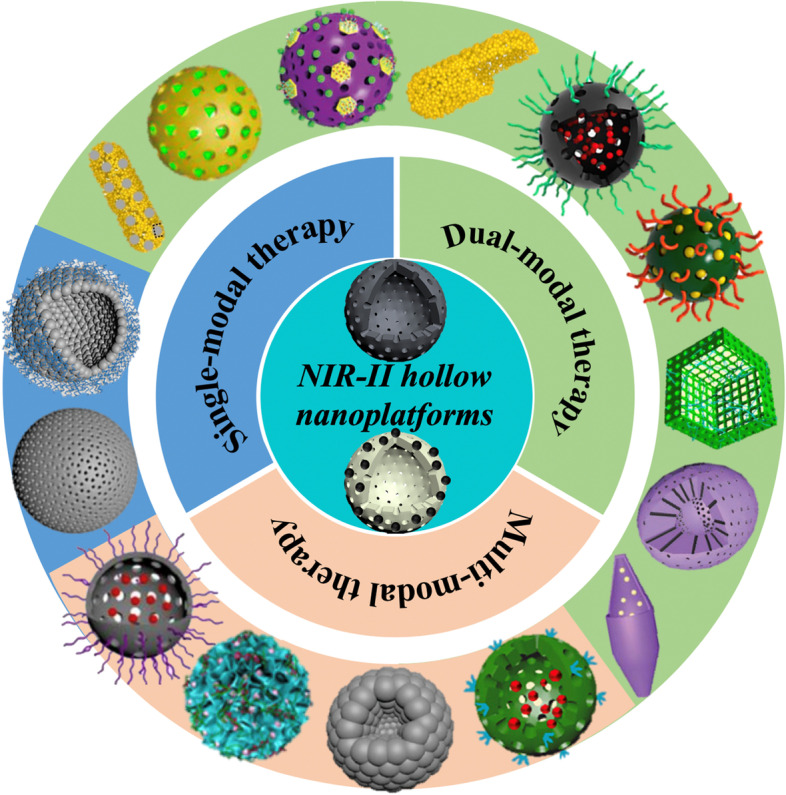
Schematic illustration of various NIR-II hollow nanoplatforms for photothermal-based therapies. There are two representative NIR-II hollow nanoplatforms described in this work, i.e. (1) hollow nanoplatforms themselves serve as NIR-II PTAs and (2) hollow nanoplatforms are decorated with NIR-II PTAs. As for the outer circle, blue, green and orange background correspond to single-modal NIR-II PTT, dual-modal NIR-II PTT-based therapies and multi-modal NIR-II PTT-based therapies. More specifically, the categories can be divided into NIR-II PTT (e.g., HPP and Ag_2_S Ve), NIR-II PTT/PDT (e.g., AuHNRs-DTPP, AAM-Ce6 and TAT-Pd@Au/Ce6/PAH/H-MnO_2_), NIR-II PTT/chemotherapy (e.g., AuHNRs-DOX, DOX-NiP PHNPs and DSF@PEG-HCuS), NIR-II PTT/catalytic therapy (e.g., PEG-Cu_2_Se HNCs and HSC-2), NIR-II PTT/gas therapy(e.g., HC-AB), PTT/CDT/chemotherapy (e.g., DOX@H-Cu_9_S_8_/PEG and HMNC), NIR-II PTT/chemo/gene therapy (e.g., CPT-RHNS-PGEA/p53) and NIR-II PTT/PDT/CDT/ST/immunotherapy (e.g., PEG-CMS@GOx). Figures were reproduced with permission from Ref. [97–111], respectively

So far, serval works have reported diverse hollow nanoplatforms or NIR-II nanoagents for cancer theranostics [38, 42, 83–96]. In this review, we for the first time present the recent progress of NIR-II hollow nanoplatforms for PTT-based cancer therapies with several sections summarized according to the treatment modalities. First of all, the synthetic methods for hollow nanoplatforms are included. Subsequently, the basic introduction of each NIR-II hollow nanoplatforms is firstly given and then a detailed description of the applications including PAI, FLI, MRI and CT imaging as well as single-modal NIR-II PTT, dual-modal NIR-II PTT-based therapies (e.g., PTT/photodynamic therapy (PDT), PTT/chemotherapy, PTT/catalytic therapy and PTT/gas therapy) and multi-modal NIR-II PTT-based therapies (e.g., PTT/chemodynamic therapy (CDT)/chemotherapy, PTT/chemo/gene therapy and PTT/PDT/CDT/starvation therapy (ST)/immunotherapy) are depicted (Scheme 1). Finally, we discuss the potential obstacles and perspectives of these novel NIR-II hollow nanotheranostics for translational applications.

## Synthetic methods for hollow nanoplatforms

### Sacrificial-template-based method

**Fig. 1:**
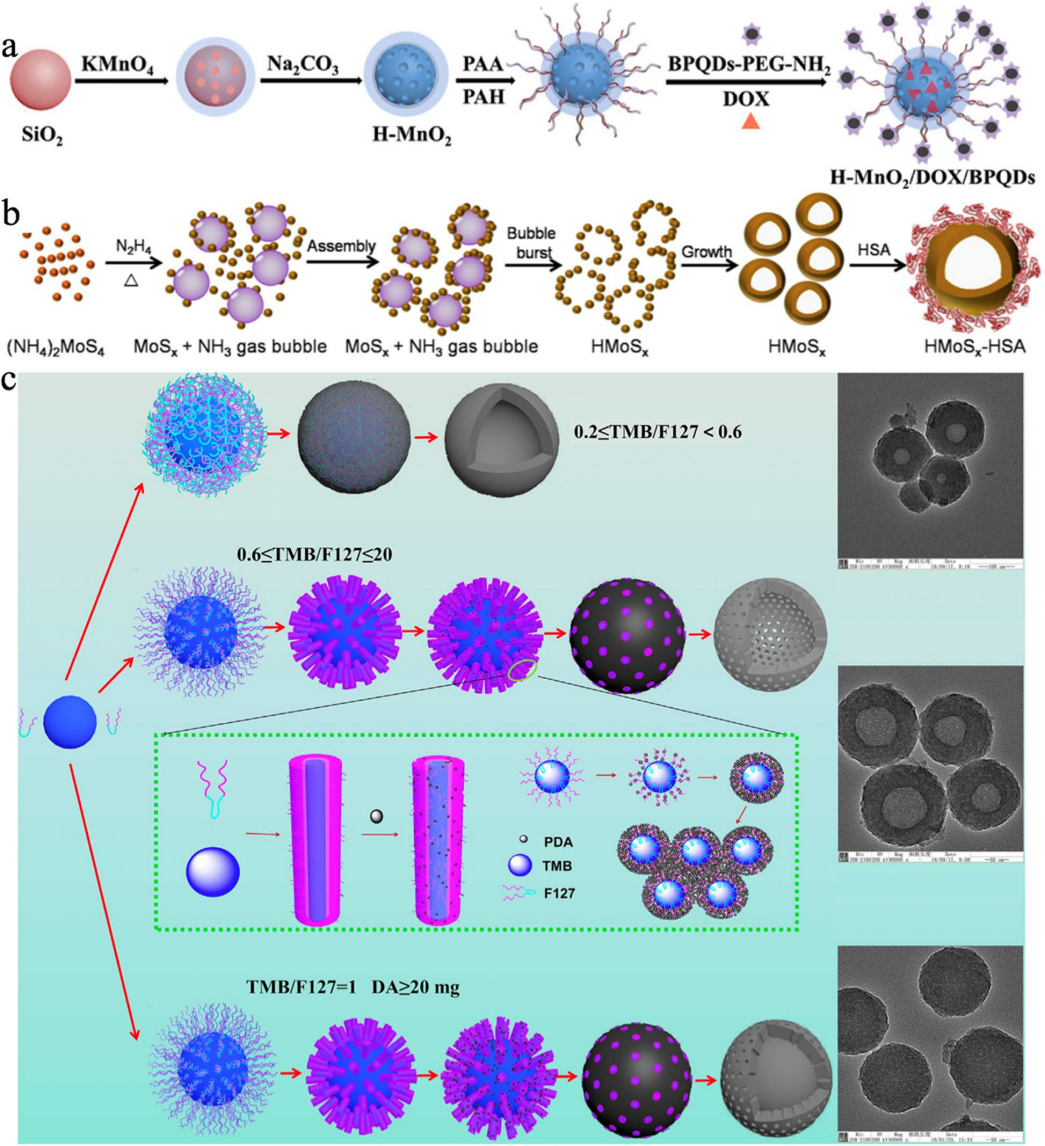
**a** Schematic illustration of nanoprobe H-MnO_2_/DOX/BPQDs synthesis route. Reproduced with permission from Ref. [120]. Copyright 2021, Wiley-VCH. **b** A scheme showing the synthesis and surface modification of HMoS_x_. Reproduced with permission from Ref. [117]. Copyright 2019, Ivyspring International Publisher. **c** Schematic illustration of the formation of PDA nanospheres (PDANSs, H-MPDANSs and MPDANSs) with different morphology (the lower left corner in the green dotted box is the longitudinal section of the columnar micelle, and the lower right corner is the cross section of the columnar micelle). Reproduced with permission from Ref. [116]. Copyright 2021, IOP Publishing Ltd

Sacrificial-template-based method is regarded as the most commonly used strategy to synthesize hollow NPs, which exploits diverse removable NPs as hard and soft templates [112, 113]. Such templates are often prepared in advance and dissolved after growing the desired materials on their surface. The dissolution of the inner templates can be realized through chemical etching or thermal decomposition, leading to highly uniform hollow NPs [114–117]. By controlling the particle size of the templates and the reaction parameters of the outer shell, the diameter and shell thickness of the resultant hollow NPs are easily tuned [118, 119]. Inorganic solid silica (SiO_2_) NPs and organic polymeric NPs have been widely explored as hard templates. For example, Wu et al. [120] utilized the unreacted organosilica on the surface of SiO_2_ NPs to react with manganese permanganate (KMnO_4_) to in situ form a uniform mesoporous manganese dioxide (MnO_2_) layer. After sodium carbonate (Na_2_CO_3_) solution treatment, the SiO_2_ was dissolved and a hollow mesoporous structured MnO_2_ (denoted as H-MnO_2_) could be obtained for dual-modal MRI/FLI-guided synergistic PTT/PDT/chemotherapy with DOX and black phosphorus quantum dots (BPQDs) co-loaded (Fig. 1a). Besides, SiO_2_ NPs have also been applied to fabricate hollow polydopamine (PDA) NPs [121], hollow mesoporous silica NPs [122], hollow carbon NPs [123] and hollow mesoporous ferric oxide NPs [124], etc. Analogously, the reduction of KMnO_4_ by poly(lactic-co-glycolic acid) (PLGA) NPs also gave rise to H-MnO_2_ NPs after etching the inner PLGA by acetone [82]. In this nanosystem, Wang and co-workers used platelet membrane (PLTM) to coat the bufalin-loaded HMnO_2_ and investigated its feasibility for cancer-specific MRI-guided combined CDT/chemotherapy. Other polymeric NPs like polystyrene NPs are also popular template for the synthesis of hollow PDA NPs [70, 125, 126]. Additionally, zeolitic imidazolate framework-8 (ZIF-8) NPs as hard templates have been involved in preparing hollow PDA NPs [127] and hollow porphyrinic metal–organic framework [114]. As for soft templates, Wang et al. [117] converted the molybdenum disulfide (MoS_2_) nanodots to hollow MoS_x_ NPs in the presence of ammonia (NH_3_) bubbles. With photosensitizer chloride aluminium phthalocyanine (AlPc) and O_2_ carrier perfluorohexane (PFH) incorporated, the resultant nanoplatform can be used for FLI/PAI/CT imaging and synergistic PTT/PDT (Fig. 1b). Lin et al. [116] adjusted the weight ratio between triblock copolymer Pluronic F127 and 1,3,5-trimethylbenzene (TMB) to fabricate hollow PDA nanospheres (H-PDANSs) and evaluated its potential as NIR laser- and acid pH-responsive DDSs. The formation mechanism of PDA NPs with different morphologies was shown in Fig. 1c. In the absence of TMB, F127 had no effect on the morphology and classical solid PDANSs were obtained. After simultaneous addition of TMB and F127, oil-in-water emulsion droplet template was formed which could result in a small amount of H-PDANSs. When the amount of TMB was sufficient (TMB/F127 ≥ 0.6), some TMB and F127 also formed a number of columnar micelles that were vertically attached to the surface of the emulsion droplets. TMB could enter into the hydrophobic interior of columnar micelles and maintain a dynamic balance between emulsion droplets and columnar micelles. Subsequently, the added DA preferentially adsorbed on the hydrophilic surface of F127 and self-polymerized to form PDA small particles, which further gathered in large quantities on the surface of the hydrophilic F127. The size of the TMB oil droplets determined the size of the cavity, and the hydrophobic cavity of the TMB/F127 columnar micelles led to the mesoporous channels. When the amount of DA was sufficiently large, it could eventually cause the PDA particles to fill with the entire TMB droplet and give rise to a complete mesoporous structure with the cavity structure disappeared.

### Self-templating method

**Fig. 2:**
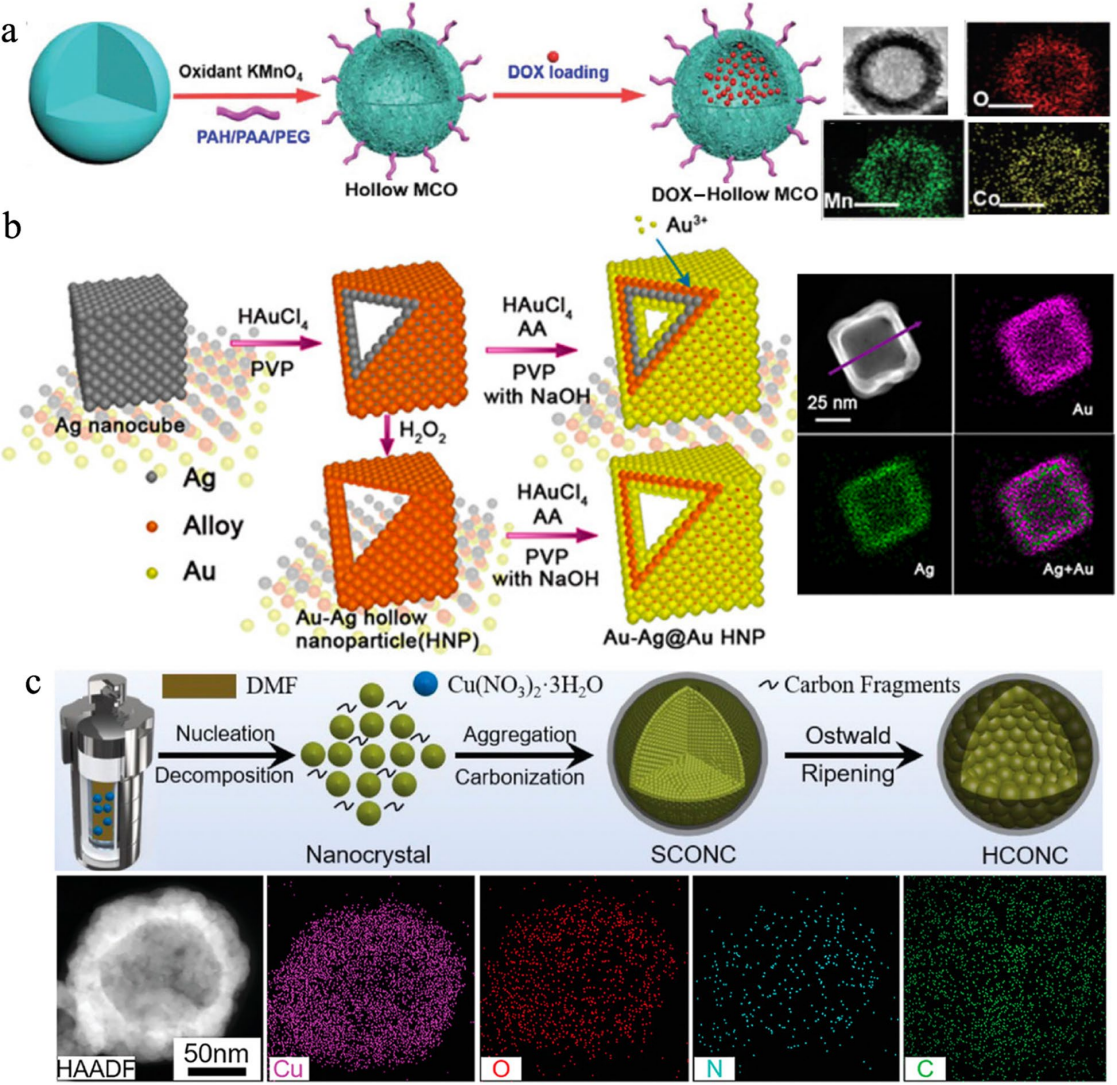
**a** The schematic illustration of MCO NP synthesis; Transmission electron microscopy (TEM) and elemental mapping image of MCO-100 NPs (diameter was ~ 100 nm). Reproduced with permission from Ref. [132]. Copyright 2021, Royal Society of Chemistry. **b** Schematic illustration of Au–Ag HNP and Au–Ag@Au HNPs; Dark-field scanning TEM (DF-STEM) images and elemental mapping images of Au–Ag@Au HNPs. Reproduced with permission from Ref. [133]. Copyright 2015, American Chemical Society. **c** Schematic illustration of the formation process of HCONC; High-angle-annular-dark-field STEM (HAADF-STEM) and corresponding element mappings of single HCONC. Reproduced with permission from Ref. [134]. Copyright 2022, Wiley-VCH.

On the other hand, self-templating method employing the transformation of self-generated internal solid NPs to hollow structures during chemical reactions, has also gained widespread attention in recent years [85, 128–131]. There are three main mechanisms involved in the self-templating method such as nanoscale Kirkendall effect, galvanic replacement reaction and Ostwald ripening process. For example, Ren et al. [132] reported a simple synthetic strategy to prepare monodisperse hollow manganese/cobalt oxide (MCO) NPs for tumor imaging and drug delivery. As shown in Fig. 2a, hollow MCO NPs were obtained from the oxidation of polyacrylic acid (PAA)-covered cobalt (Co) by KMnO_4_, in which the formation of hollow cavities could be attributed to the Kirkendall effect, i.e. the different diffusion rates of MnO_4_^−^ and Co atoms led to the pore generation. Jiang et al. [133] fabricated gold − silver@gold (denoted as Au − Ag@Au) hollow NPs with improved chemical stability and enhanced PCE of 36.5% at 808 nm by the replacement reaction for effective destruction of MCF-7 breast cancer cells (Fig. 2b). Meng et al. [134] developed hollow cuprous oxide@nitrogen-doped carbon (denoted as HCONC) with dual-shell structures via a one-step hydrothermal method based on cupric nitrate and dimethyl formamide for GSH-depletion boosted CDT (Fig. 2c). The preparation of HCONC mainly contained three stages including (1) the nucleation of cuprous oxide (Cu_2_O) nanocrystals under high temperature and pressure as well as dissolved oxygen; (2) the formation of primary solid Cu_2_O nanoclusters by aggregation with SCONC; (3) the further growth of outermost nanocrystals and consumption of small internal particles during Ostwald ripening gave rise to a hollow cavity and generated HCONC.

Though the sacrificial-template-based approach can form various hollow NPs with uniform morphology, tunable diameter and shell thickness, it often requires strong acid and alkali as well as harmful organic solvents [135, 136]. As a contrast, the self-templating method possesses simplified synthetic procedures and reduced chemical waste formation [137–141]. Both of the two strategies have been widely explored for constructing hollow nanoplatforms for highly efficient NIR-II PTT. Such nanoplatforms themselves can be promising NIR-II PTAs or facilitate the growth of NIR-II PTAs on their surfaces [97, 101]. More importantly, the hollow cavity is able to encapsulate various diagnostic and therapeutic components, displaying tremendous promise in bioimaging, drug delivery and tumor therapy [118, 142]. In the following sections, diverse nanoplatforms are introduced for single NIR-II PTT, NIR-II PTT-based dual-modal therapies including PTT/PDT, PTT/chemotherapy, PTT/catalytic therapy and PTT/gas therapy as well as NIR-II PTT-based multi-modal therapies such as PTT/CDT/chemotherapy, PTT/chemo/gene therapy and PTT/PDT/CDT/ST/immunotherapy. At the meanwhile, their capacities in FLI, MRI, PAI and CT imaging are also incorporated.

## Hollow nanoplatforms for single NIR-II PTT

Thanks to the merits of non-invasiveness, efficient tumor ablation and low systemic toxicity, PTT excited and controlled by NIR light has been regarded as a promising candidate for the treatment of different types of cancers [143–145]. Currently, most of the PTT are conducted under an 808 nm laser irradiation, in which the low MPE value and limited penetration depth still hinder their further applications. Therefore, the development of NIR-II PTAs has become more and more popular [44, 90, 93, 146, 147]. Though organic nanomaterials especially conjugated polymers prove to be excellent NIR-II PTAs, the corresponding hollow structures are seldom reported [86]. So past studies have focused mainly on the design of various inorganic NIR-II hollow nanoplatforms including carbon-based, Au-based and metal chalcogenide nanomaterials, etc. [99, 100, 102, 105–107, 148].

For example, Xu et al. [97] successfully prepared a polyethylene glycol-graft-polyethylenimine (PEG-g-PEI) modified hollow carbon nanosphere (denoted as HPP) using SiO_2_ NPs as the templates. The uniform HPP (~ 215 nm) possessed a high PCE of 45.1% at 1064 nm due to the excellent optical absorbance in the NIR-II biowindow, and the outer PEG-g-PEI functionalization guaranteed its aqueous dispersity and biocompatibility. In vitro and in vivo therapeutic outcomes were evaluated with a safe laser power density (1064 nm, 0.6 W/cm^2^), showing that the HPP exhibited remarkable photocytotoxicity towards 4T1 cells and tumors. Only 10% of the cells survived when the concentration was 80 µg/mL and the tumor sizes were dramatically diminished with three groups completely eradicated after HPP + 1064 nm laser treatment.

**Fig. 3:**
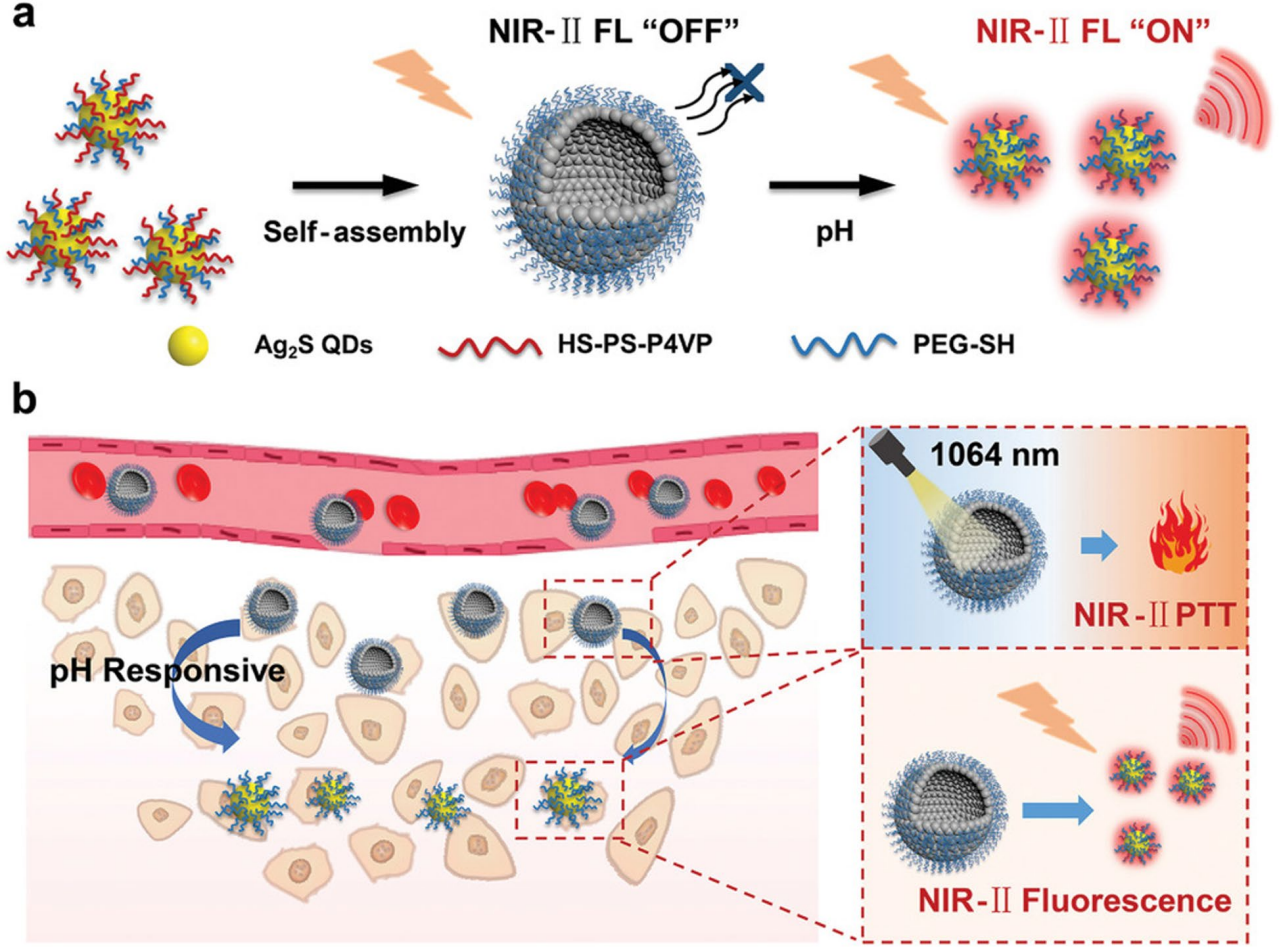
Illustration of (**a**) self-assembly of Ag_2_S QDs into stimulation-responsive Ag_2_S Ve and (**b**) Biomedical applications of Ag_2_S Ve for pH-activated NIR-II FLI guided-NIR-II PTT. The disassembly-induced in situ controllable activated NIR-II fluorescence recovery resulted a real-time monitoring for deep-penetration toward solid tumors. Reproduced with permission from Ref. [98]. Copyright 2021, Wiley-VCH.

Liu et al. [98] fabricated a smart tumor microenvironment (TME)-activatable hollow silver sulfide vesicle (denoted as Ag_2_S Ve) for NIR-II FLI-guided NIR-II PTT. In this design, ultrasmall Ag_2_S QDs (~ 8 nm) were first synthesized in dimethyl formamide (DMF) with pH-sensitive thiolated polystyrene-co-poly(4-vinylpyridine) (HS-PS-P4VP) and hydrophilic poly(ethylene glycol)-thiol (PEG − SH) polymers modified and subsequently dispersed in chloroform after discarding DMF. When sodium aqueous dodecyl sulfate (SDS) solution was added, the Ag_2_S QDs could be transformed to Ag_2_S Ve via a self-assembly procedure under sonication. With the removal of chloroform, the final Ag_2_S Ve aqueous dispersion was obtained. Interestingly, the larger particle size (160 nm) of Ag_2_S Ve gave rise to enhanced tumor accumulation. Due to the responsiveness of PS-P4VP at acidic TME, the hydrophobic copolymer was changed to hydrophilic to allow the release of PEG-SH modified Ag_2_S QDs, turning on the remarkable NIR-II fluorescence. Meanwhile, the NIR absorption enabled the Ag_2_S QDs to be a good NIR-II PTA. Such novel TME-activated theranostic features were further investigated in vivo for efficient and accurate NIR-II FLI-guided PTT of subcutaneous 4T1 tumors, displaying enormous potential for avoiding health tissues from photothermal damage as well (Fig. 3).

## Hollow nanoplatforms for NIR-II PTT-based dual-modal therapies

Although tremendous progress has been made, it is still quite difficult to realize a complete cure via single-modal NIR-II PTT as the unique TME promotes the proliferation and metastasis of cancers [149–151]. Besides, PTT has been demonstrated to be restricted by the overexpressed heat shock proteins (HSPs) found in serval types of cancers [35, 152, 153]. To this end, synergistic treatment as a new paradigm proves to be the hotspot not only in clinical judgment but also scientific research, aiming at significantly improving the antitumor efficacy [14, 84, 154]. Most notably, reactive oxygen species (ROS) accumulation is able to reduce the HSPs expression and in turn, hyperthermia generated from PTT can boost tumor blood flow, which is beneficial for O_2_-dependent cancer therapies [155–157]. This means that the combination of NIR-II PTT and ROS-mediated treatment modalities such as PDT, chemotherapy and catalytic therapy can realize super-additive effects. In addition, gas therapy with different kinds of gaseous signaling molecules included is also capable of inhibiting the inflammation during PTT to greatly enhance the therapeutic outcomes [158, 159]. In this section, we will introduce these dual-modal treatment strategies in detail.

### PTT/PDT

As another kind of phototherapy, PDT integrating the merits including noninvasiveness, ideal tumor-destroying selectivity, low side effects and insignificant drug resistance has also been widely investigated and demonstrated to be a promising candidate for cancer therapy [160, 161]. Briefly, PDT kills cancer cells by generating cytotoxic ROS through laser irradiating diverse photosensitizers in the presence of O_2_ [162, 163]. Nowadays, the majority of photosensitizers involved in PDT are small organic molecules (e.g., chlorin e6 (Ce6) [164], indocyanine green (ICG) [165] and methylene blue (MB) [166], etc.), which exhibit the drawbacks of non-specific biodistribution and damage to skin as well as other normal tissues after administration. In light of these, hollow NIR-II PTAs can serve as smart nanocarriers to load photosensitizers and control their functions on demand.

**Fig. 4:**
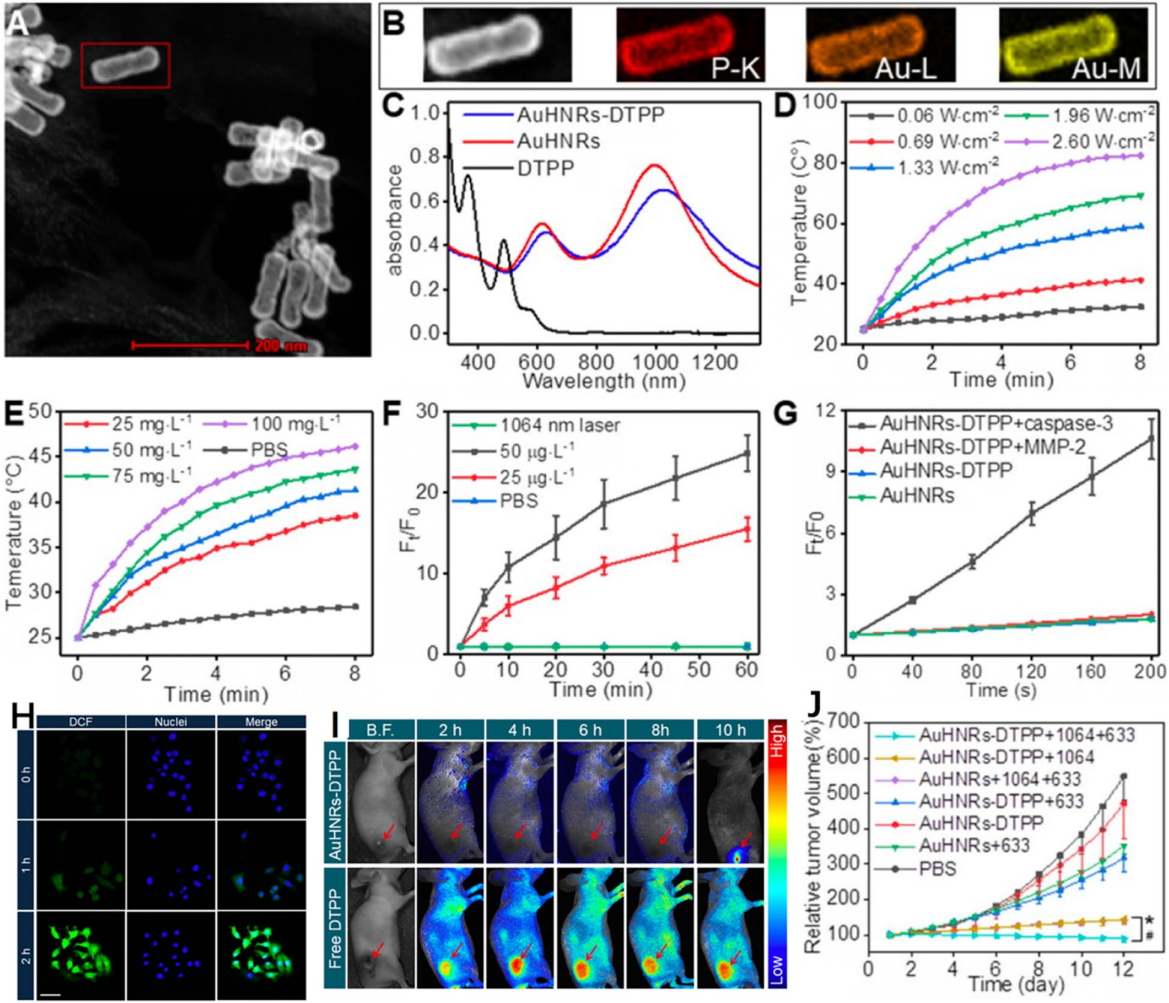
**A** STEM imaging of AuHNRs-DTPP. **B** HAADF-STEM imaging and element mapping (using P-K shell, Au-L shell and Au-M shell) of AuHNRs-DTPP. **C** Ultra-violet and visible (UV-Vis) spectrum of AuHNRs, DTPP and AuHNRs-DTPP. Temperature change of AuHNRs-DTPP in PBS (**D**) at the concentration of 50 g L^–1^ AuHNRs-DTPP with various laser power and (**E**) under the laser power of 0.69 W cm^–2^ with various concentration. **F** Relative fluorescence intensity changes of AuHNRs-DTPP incubated with recombinant caspase-3. 10 min irradiation with 1064 nm laser (0.69 W cm^–2^) was taken as control. **G** ROS generation of AuHNRs-DTPP (treated with recombinant caspase-3 for 2 h) under light irradiation using 2′-7′-dichlorofluorescin diacetate (DCFH-DA) as the sensor. AuHNRs, PBS and matrix metalloproteinase-2 (MMP-2) were used as controls. **H** Confocal laser scanning microscopy (CLSM) imaging of intracellular ROS formation under 633 nm laser. **I** In vivo fluorescence images of tumor-bearing mice after intravenous injection of AuHNRs-DTPP, free DTPP was used as control, 1064 nm laser irradiation was conduct at 8th h. **J** Relative tumor volume in different groups. Reproduced with permission from Ref. [99]. Copyright 2019, Ivyspring International Publisher

For example, Zhang et al. [99] developed a hollow Au nanorods-based nanoplatform (denoted as AuHNRs-DTPP) for NIR-II PTT, supplementary PDT and real-time apoptosis FLI. The nanoplatform was consisted of Au hollow nanorods (AuHNRs) and photosensitizer containing chimeric peptide PpIX-PEG8-GGK(TPP)GRDEVDGC (abbreviated as DTPP, PpIX was short for protoporphyrin), in which the AuHNRs were obtained using tellurium (Te) nanorods as the template and the DTPP was prepared via a solid-phase peptide synthesis method. As shown in Fig. 4a, b, the AuHNRs displayed typical hollow structure and the length/width ratio was calculated to be 3 : 1. After loading DTPP onto the AuHNRs, the plasmon resonances peaks (614 and 990 nm) of AuHNRs showed obvious red-shift while the absorption peaks of DTPP were quenched (Fig. 4c). The intensive NIR-II absorption provided excellent photothermal conversion capacity for AuHNRs-DTPP as NIR-II PTA. As shown in Fig. 4d, e, the temperature of the AuHNRs-DTPP dispersion went up with the increase of laser power density, time and concentration. The quenched fluorescence and inhibited ROS production of AuHNRs-DTPP were capable of protecting the non-tumoral tissues from photodynamic toxicity. Interestingly, the presence of human recombinant caspase-3 could cleave the linkage between AuHNRs and DTPP, leading to the release of DTPP and thus the recovery of fluorescence as well as the ROS-generating ability upon a 633 nm laser irradiation (Fig. 4f, g). Similar trends were found at the cellular level and the vivid green fluorescence confirmed the potent PDT effects (Fig. 4 h). For in vivo experiments, the AuHNRs-DTPP could accumulate at the tumor site via enhanced permeability and retention (EPR) effect after intravenous injection. Compared to the non-specific distribution of free DTPP, the AuHNRs-DTPP treatment gave limited fluorescence at 8 h post-injection but the fluorescence signal was significantly strengthened with the assistance of 1064 nm laser irradiation. These data evidenced that the NIR-II PTT could facilitate the PDT and remarkable tumor suppression was achieved owing to the well-integrated PTT/PDT (Fig. 4i, j).

It is known that PDT relies greatly on the O_2_ level, but TME is often hypoxic [163, 167]. Nowadays, two main methods (1) direct transportation of O_2_ by perfluorocarbon nanodroplets or oxygenated hemoglobin, etc. and (2) in situ catalytic generation of O_2_ using catalase and catalase-mimicking nanozymes are applied to improve the intratumoral O_2_ concentration [168, 169]. The latter one becomes more popular on account of the overproduced endogenous hydrogen peroxide (H_2_O_2_), and MnO_2_ is one of the most appealing catalase-mimicking nanozymes [170, 171]. For example, Wu et al. [100] fabricated a novel multifunctional Au/Ag-MnO_2_-Ce6 hollow nanospheres (denoted as AAM-Ce6 HNSs) for PAI/FLI/MRI-guided PTT/PDT. The hollow structured Au/Ag alloy was first prepared via a galvanic replacement reaction, followed by in situ growth of MnO_2_ NPs on the surface with KMnO_4_ added. After Ce6 loading and SH-PEG modification, the final AAM HNSs were obtained. The AAM-Ce6 HNSs displayed broad absorption in the NIR region and outstanding NIR-II photothermal effects (PCE = 52.5% at 1064 nm), which could allow excellent PAI and PTT. The outer MnO_2_ NPs showed rapid responsiveness towards TME, decomposing endogenous H_2_O_2_ to produce O_2_ for hypoxia-relieved PDT and simultaneously releasing abundant Mn^2+^ ions as MRI CAs. With the FLI performance of Ce6 integrated, triple-modal imaging was realized to guide the combined PTT/PDT. In vitro and in vivo experiments revealed that the synergistic therapeutic efficacy was much better than any single-modal treatment, showing bright prospect for cancer theranostics.

**Fig. 5:**
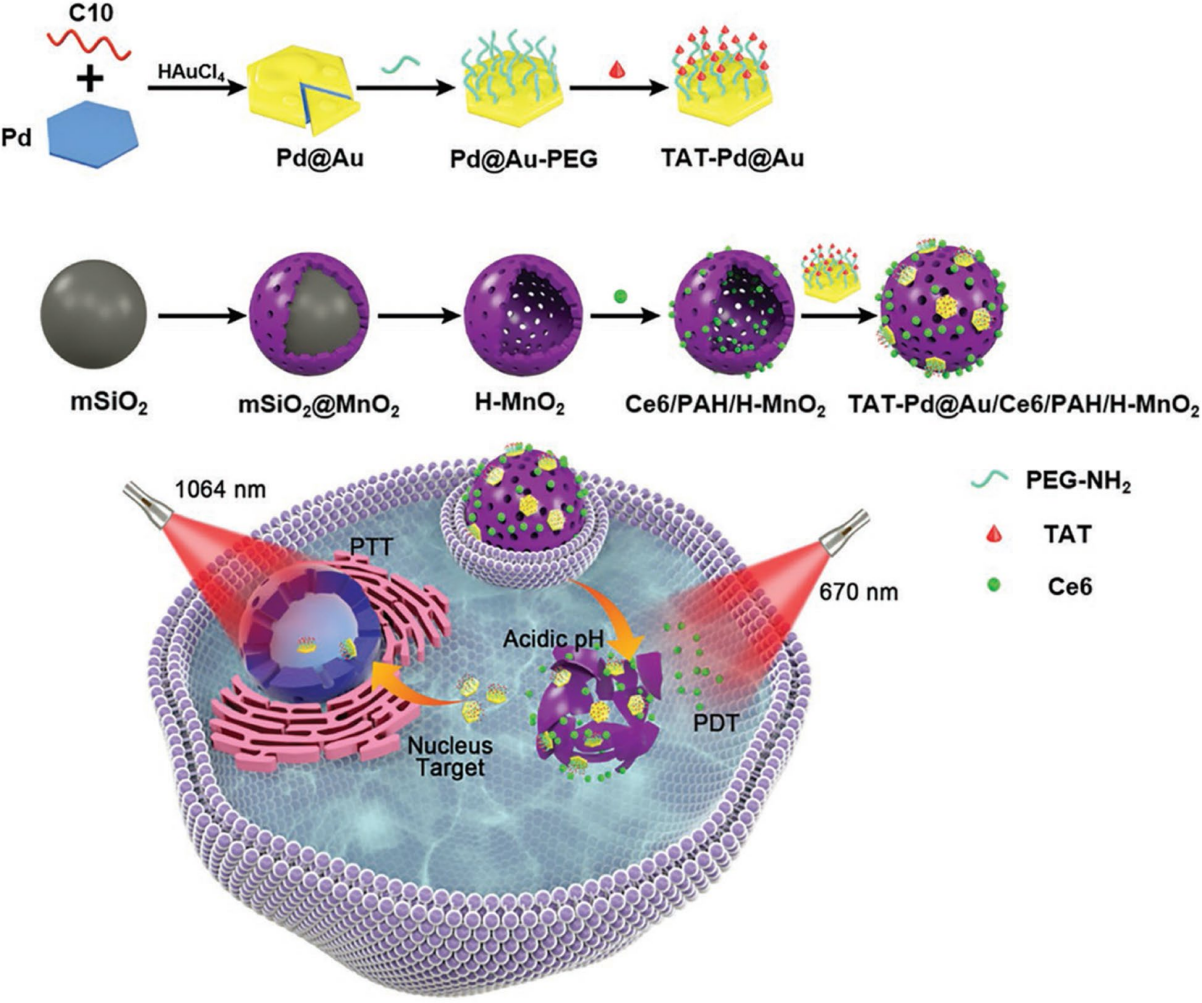
Preparation procedure of the TAT-Pd@Au@Ce6/PAH/H-MnO_2_ and schematic illustration of TAT-Pd@Au/Ce6/H-MnO_2_ for nucleus-targeted PTT and hypoxia-relieved PDT. Reproduced with permission from Ref. [101]. Copyright 2020, Wiley-VCH.

In other work, Zhang et al. [101] constructed a multifunctional nanoplatform (denoted as TAT-Pd@Au/Ce6/PAH/H-MnO_2_, Pd was short for palladium) by decorating transactivator of transcription (TAT)-Pd@Au nanoplates onto hollow mesoporous MnO_2_ (H-MnO_2_), in which the H-MnO_2_ was loaded with Ce6 and modified with a cationic polymer poly allylamine hydrochloride (PAH) layer (Fig. 5). The PEG-TAT functionalization endowed the nanoplatform with long blood circulation time and nuclear targeting ability. Once reaching the tumor site, the inner H-MnO_2_ could be degraded by the acidic H_2_O_2_ to produce Mn^2+^ for MRI and O_2_ to alleviate the hypoxic atmosphere for enhanced PDT, respectively. At the meanwhile, the released small TAT-Pd@Au nanoplates were able to effectively enter into the nucleus to conduct NIR-II PTT with an outstanding PCE up to 56.9%. Consequently, significant therapeutic effects could be achieved due to the synergistic PTT/PDT. This novel nanotheranostics inspires a new strategy for subcellular targeting cancer diagnosis and therapy.

### PTT/chemotherapy

Chemotherapy using different kinds of chemotherapeutic drugs has been an essential part for clinical cancer treatment over the past decades [172–174]. However, the deficient tumor accumulation and overdose of drugs in current chemotherapy often lead to poor curative outcomes and severe side effects [175–177]. Therefore, a large number of smart DDSs including polymeric NPs [178], SiO_2_ NPs [179] and metal-based NPs [5, 180], etc. are developed, which can response to the unique TME such as low pH [181], high glutathione (GSH) [182] and enzyme [183], etc., as well external triggers like photo [184] and ultrasound [185], etc. for site-specific drug release with higher tumor accumulation and reduced systemic toxicities. Among the reported chemotherapeutic drugs, DOX is one of the most commonly explored model drugs and demonstrated to effectively treat breast carcinoma and ovarian carcinoma, etc. [186, 187].

By utilizing a selenium (Se)-doped Te nanorod as the template, Cai et al. [102] synthesized anisotropic AuHNRs with tunable aspect ratios. The AuHNRs displayed a LSPR peak in the NIR-II area when the aspect ratio was ~ 3, which was much less than that of the conventional Au nanorods. Upon a 1064 nm laser irradiation for 10 min, the temperature of the AuHNRs aqueous dispersion (0.15 mg mL^− 1^) increased by about 35 °C and the PCE was calculated to be 33%. Moreover, AuHNRs proved to be nontoxic and able to encapsulate DOX, in which the loading efficiency went up with the elevation of feeding amount. Based on these features, the AuHNRs could serve as ideal nanoagents for combined PTT/chemotherapy. Furthermore, the feasibility of AuHNRs as multifunctional CAs in PAI and CT imaging were also evidenced.

In order to resolve the nonbiodegradability and potential long-term toxicities of inorganic PTAs, Liu et al. [103] successfully fabricated acidic/oxidative double switch degradable and clearable bovine serum albumin (BSA)-modified trinickel monophosphide (NiP) porous hollow nanospheres (denoted as NiP PHNPs). On account of the excellent NIR-II absorption, the NiP PHNPs possessed a remarkable PCE of 56.8% and high molar extinction coefficient (1.577 × 10^10^ M^− 1^ cm^− 1^) at 1064 nm. Such superior photothermal effect led to highly effective PTT with the tumors being completely eliminated. Interestingly, the hollow structure provided the capacity of NiP PHNPs for DOX delivery, while the acidic and oxidative degradation behaviors could trigger the on-demand release of loaded DOX in TME. Benefiting from the PTT/chemotherapy, the tumors were fully eradicated without recurrence. Additionally, the good paramagnetic and high molar extinction coefficient properties enabled the NiP PHNPs to be T_1_-weighted MRI and PAI CAs for diagnosis of tumors and guiding the therapeutic process. This work inspires the researchers to broaden the biomedical applications of transition metal phosphides.

**Fig. 6:**
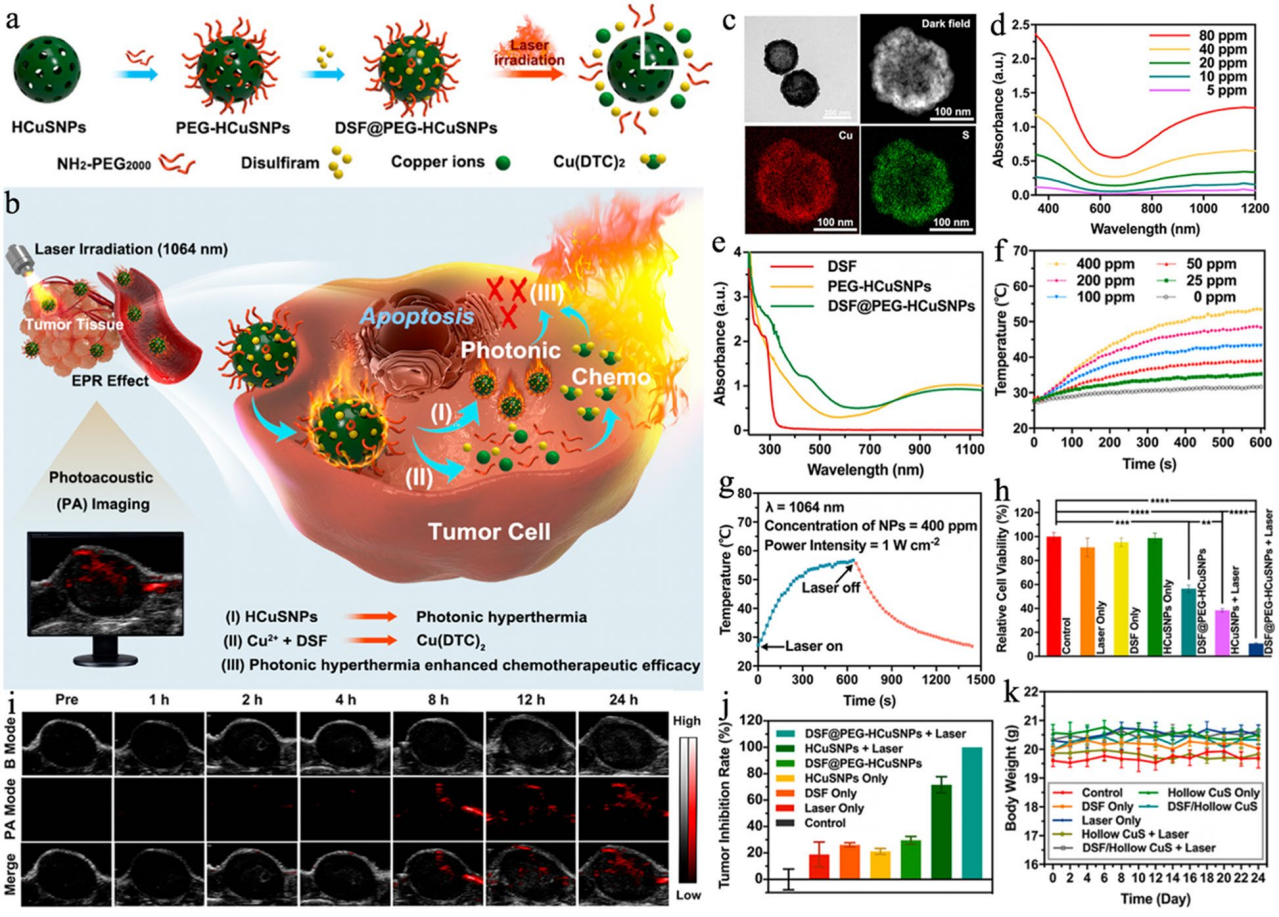
Schematic illustration of (**a**) the fabrication of DSF@PEG-HCuSNPs and (**b**) the corresponding synergy of NIR-II-induced photonic hyperthermia and the in situ formation of a Cu(DTC)_2_ complex for augmented chemotherapeutic efficacy of DSF-based chemotherapy. **c** TEM images and elemental distribution mappings of HCuSNPs. **d** UV–vis–NIR absorption spectra of varying doses of HCuSNPs (5, 10, 20, 40, and 80 ppm). **e** UV–vis–NIR absorption spectra of DSF (50 ppm), PEG-HCuSNPs (60 ppm), and DSF@PEG-HCuSNPs (60 ppm). **f** Heating curves of varying concentrations of DSF@PEG-HCuSNPs upon 1064 nm laser exposure (1 W cm^–2^). **g** Photo-to-heat conversion capability of a DSF@PEG-HCuSNPs aqueous dispersion upon 1064 nm laser exposure (1 W cm^–2^). **h** Relative cellular survival rates after varied treatments, including control, DSF only, HCuSNPs only, NIR-II laser only, DSF@PEG-HCuSNPs, HCuSNPs + NIR-II laser, and DSF@PEG-HCuSNPs + NIR-II laser groups. **i** PA images in vivo in tumor tissues after intravenous administration of DSF@PEG-HCuSNPs at varying time intervals. **j** Tumor inhibition rate and **k** Body weight of 4T1 tumor-bearing nude mice in the control, NIR-II laser only, DSF only, HCuSNPs only, DSF@PEG-HCuSNPs, HCuSNPs + NIR-II laser, and DSF@PEG-HCuSNPs + NIR-II laser group. Reproduced with permission from Ref. [104]. Copyright 2021, American Chemical Society

In addition, Shi group revealed that disulfiram (DSF) could be an efficient chemotherapeutic agent after chelation with Cu^2+^ ions (denoted as Cu(DTC)_2_). Actually, DSF is a U.S. Food and Drug Administration (FDA)-approved drug for chronic alcoholism treatment, this method tends to be quite appealing because it in situ converts the nontoxic DSF to toxic Cu(DTC)_2_ in the tumor site. Based on that, Liu et al. [104] loaded DSF into NH_2_ − PEG_2000_-modified hollow CuS NPs (denoted as DSF@PEG-HCuS NPs) for NIR-II PTT and chemotherapy (Fig. 6a, b). As shown in Fig. 6c, the HCuS NPs were hollow and spherical in morphology, possessing an average particle size of 220 nm. UV–vis–NIR spectra evidenced the strong optical absorption of HCuS NPs especially in the NIR-II area with an extinction coefficient calculated to be 15.69 L g^− 1^ cm^− 1^ at 1064 nm (Fig. 6d). Besides, the growing absorbance from 250 to 325 nm in the green curve suggested that the DSF was successfully loaded as DSF exhibited pronounced absorption within this range (Fig. 6e). Upon a 1064 nm laser irradiation, the DSF@PEG-HCuS NPs dispersions showed obvious temperature elevation. Specifically, the temperature increased from 27.9 to 53.5 °C at a concentration of 400 ppm after irradiation for 10 min, demonstrating that the DSF@PEG-HCuS NPs could be a good NIR-II PTA (PCE = 23.8%) (Fig. 6f, g). After internalization by 4T1 cells, the DSF@PEG-HCuS NPs underwent low pH-triggered degradation that rapidly promoted the DSF and Cu^2+^ ions release, resulting in the generation of cytotoxic Cu(DTC)_2_. The DSF-based chemotherapy was evidenced by the high percentage (55.3%) of cell death that could be further enhanced by the NIR-II PTT, and the synergistic NIR-II PTT/chemotherapy led to more efficient cell-killing effects (89.3%) (Fig. 6 h). Owing to the good photothermal capability, the DSF@PEG-HCuS NPs also served as the PAI CAs to guide the treatment process. As shown in Fig. 6i, the PA signal at the tumor site amplified over time and reached the maximum at 24 h post-injection. In vivo curative effects were in line with the in vitro results as expected, the DSF@PEG-HCuS NPs + 1064 nm laser treatment induced an inhibition rate of 100%, in which the 4T1 tumors were totally eliminated and no further recurrence and significant body weight changes were found throughout the 24-day period (Fig. 6j, k). This work presents a distinctive strategy of Cu^2+^ complexation-triggered nontoxicity to toxicity conversion for photothermal/DSF-based chemotherapy.

### PTT/catalytic therapy

Catalytic therapy is an emerging treatment paradigm that attracts great attention in recent years [188–191]. It utilizes typical endogenous substances to realize tumor-specific therapy with high catalytic activity and negligible side effects [192, 193]. Up to now, CDT [194–196], glucose oxidase (GOx)-based therapy [197–199] and various nanozymes-instructed therapies have been widely investigated in cancer catalytic treatments. Among them, CDT based on Fenton or Fenton-like reactions is most popular, which employs diverse transition metal ions (e.g., Fe^2+^, Co^2+^, Cu^+^, and Mn^2+^, etc.) to catalyze intracellular H_2_O_2_ to produce toxic hydroxyl radical (•OH) [200, 201]. However, the CDT efficacy is limited due to the mild pH and deficient amount of H_2_O_2_ [202, 203]. In light of this, PTT is commonly bundled with CDT since the hyperthermia can promote the Fenon/Fenton-like reaction rates, while the •OH also can attack HSPs to overcome the heat-resistance during PTT, leading to remarkably enhanced therapeutic effects [194, 204, 205].

**Fig. 7:**
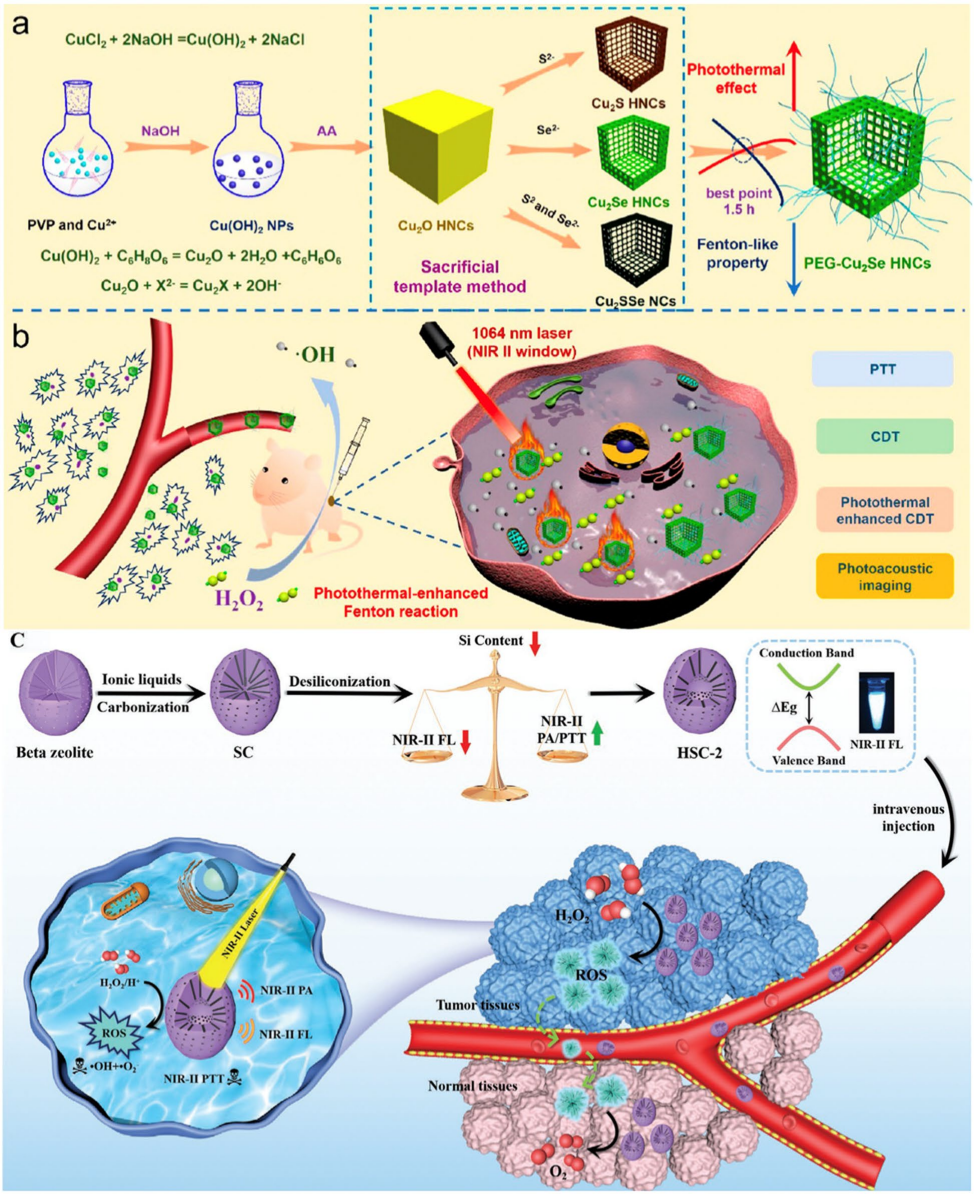
Schematic Illustration of (**a**) Preparation process of PEG-Cu_2_Se HNCs. **b** Proposed synergistic antitumor mechanism of PEG-Cu_2_Se HNCs for photothermal-enhanced CDT in NIR II Window. Reproduced with permission from Ref. [105]. Copyright 2019, American Chemical Society. **c** Schematic illustration of the adjustable photoacoustic/fluorescence imaging-guided photothermal/catalytic therapy in NIR-II window. Reproduced with permission from Ref. [106]. Copyright 2021, Wiley-VCH.

For example, Wang et al. [105] proposed an anion exchange method using Cu_2_O nanocubes (NCs) as the template to prepare cuprous selenide (Cu_2_Se) hollow HNCs for synergistic PTT/CDT (Fig. 7a, b). It was noticeable that the NIR-II absorption of the system gradually strengthened but the Fenton-like performance gradually weakened during the transforming process from Cu_2_O NCs to Cu_2_Se HNCs. After reaction for 1.5 h, the optimized Cu_2_Se HNCs exhibited both outstanding PCE (50.89% at 1064 nm) in the NIR-II biowindow and satisfied Fenton-like property. Subsequent SH-PEG modification enabled the final PEG-Cu_2_Se HNCs to possess good water dispersibility, stability and biocompatibility. In vitro experiments indicated that PEG-Cu_2_Se HNCs were able to catalyze H_2_O_2_ to generate abundant •OH via Fenton-like reaction for effective cancer cells apoptosis. Under a 1064 nm laser irradiation, the cancer cells could be completely eliminated due to the fact that the mild hyperthermia generated from the photothermal process was capable of accelerating the Fenton-like reaction to realize a synergistic manner. In vivo investigations also demonstrated the remarkable antitumor efficiency of combined PTT/CDT, which was more prominent than that of single PTT or CDT. This study offers a new method for designing copper-based CDT agents as well as evidences the tremendous potential of such multifunctional nanoagent with photothermal-boosted CDT efficacy for cancer theranostics.

Benefiting from the high stability, low cost and ease of preparation, nanozymes that mimic both the unique physicochemical performances of nanomaterials and catalytic properties of natural enzymes have also become the research focus in cancer theranostics [206–209]. In recent years, MnO_2_ as a catalase-like nanozyme is involved in numerous nanotheranostics to overcome hypoxia via decomposing endogenous H_2_O_2_ [210, 211]. Besides, Au NPs prove to be good GOx-like nanozyme that deplete intratumoral glucose for ST [212, 213]. Nowadays, it is found that one single nanozyme can be equipped with multifunctionality to amplify the curative effects [153, 214]. For example, Zheng et al. [106] designed and constructed a NIR-II PAI/NIR-II FLI-tunable zeolite–carbon-based nanozyme (denoted as HSC-2) for precisely dual-modal imaging-guided synergistic photothermal-catalytic therapy (Fig. 7c). Zeolite nano-Beta with three dimensional 12-ring pore system and large surface area was first selected as the matrix, the electronic structure of which could be transformed from the indirect to direct band gap by carbon doping due to the adsorption capability of ionic liquids, resulting in excellent NIR-II FLI performance. Interestingly, the etching process of silicon gave rise to remarkable dual-modal NIR-II PAI/NIR-II FLI properties that was facilitated by optimizing silicon/carbon ratio, concurrently guaranteeing effective PTT in the NIR-II biowindow. The PCE and extinction coefficient of HSC-2 were about 41.41% and 2.01 L g^− 1^ cm^− 1^, respectively, while the quantum yield (QY) of HSC-2 in water was calculated to be 0.412% using IR-1061 in dichloromethane (QY = 1.7%) as a reference. More importantly, the HSC-2 exhibited typical peroxidase-mimicking activity in TME, which was able to produce •OH and superoxide anion (•O_2_^−^) by catalyzing intratumoral H_2_O_2_ to increase the oxidative stress for cancer treatment. Moreover, the catalytic process could be further significantly promoted by the photothermal effect, leading to prominent antitumor efficacy under NIR-II PAI/FLI guidance. Additionally, the catalase-like property of HSC-2 was capable of eliminating excessive ROS in the normal cells to protect them from oxidative damage. Such all-in-one nanozymes provides a new dimension for accurate and efficient cancer theranostics.

### PTT/gas therapy

Gas therapy has been considered as a green and promising therapeutic modality via applying high concentration of gaseous drugs such as hydrogen (H_2_), nitric oxide (NO) and carbon monoxide (CO), etc.) [215–217]. Though excess gaseous drugs are beneficial for treating cancers, the release manners of them should be precisely controlled to reduce the harm to healthy tissues [218, 219]. Previous studies have evidenced that PTT is able to destroy the integrity of cell membrane to trigger the leakage of intracellular ROS and induce proinflammatory reactions, leading to tumor regeneration and spread [220]. Therefore, inhibiting inflammation appears to be a potent choice to greatly improve the PTT efficacy. Among these gasotransmitters, H_2_ shows great potential in disturbing the redox homeostasis by scavenging ROS, thus alleviating the oxidative stress-mediated inflammatory tissue injury [221, 222]. The combination of PTT and H_2_ gas therapy may be of significant potential in achieving remarkably synergistic treatment outcomes.

**Fig. 8:**
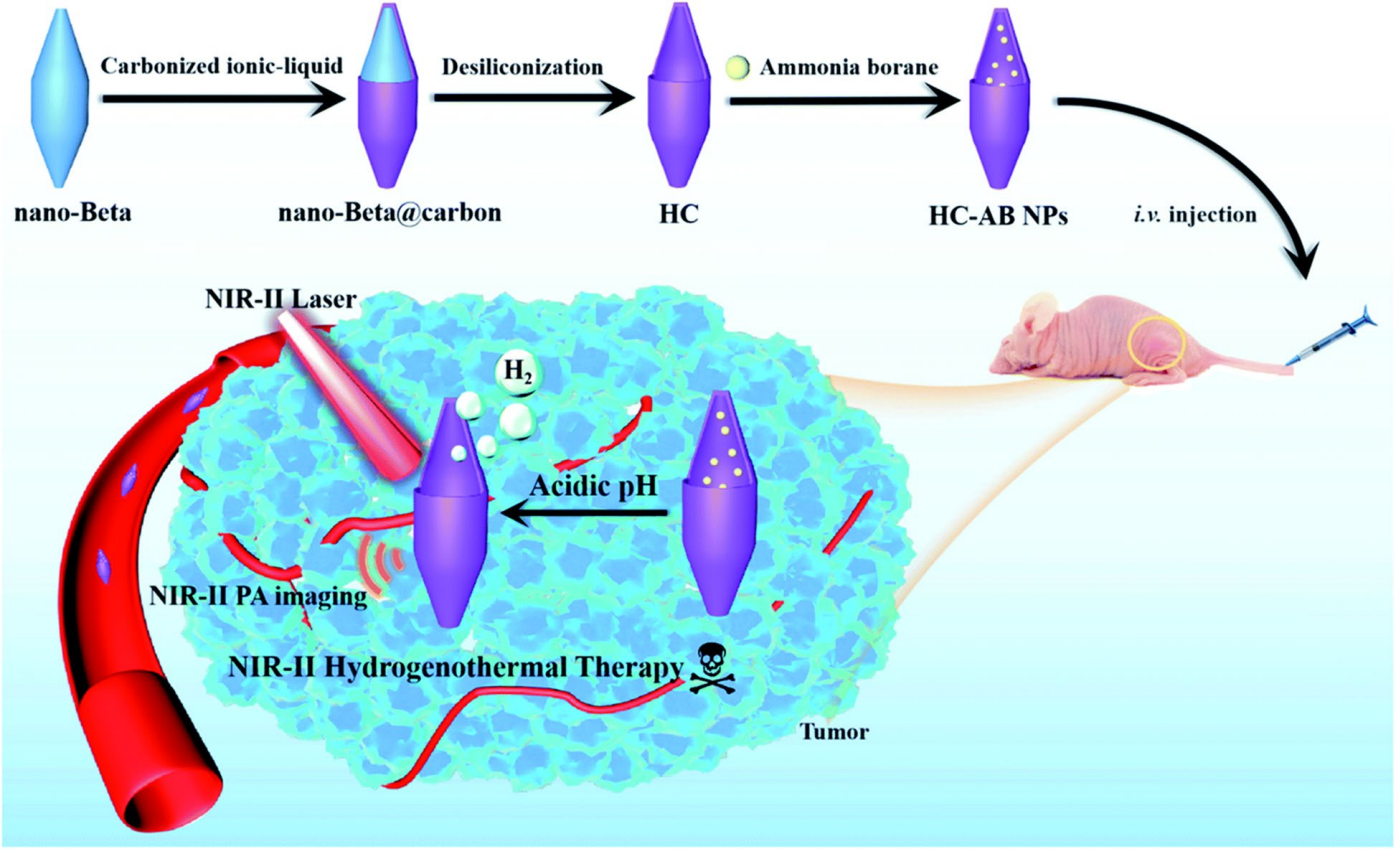
Schematic illustration of preparation of HC-AB NPs and their therapeutic mechanism. Reproduced with permission from Ref. [107]. Copyright 2021, Royal Society of Chemistry

Ammonia borane (AB) is a commonly used pH responsive H_2_ donor and it has been reported that the incorporation of AB with different PTAs is capable of promoting PTT and mitigating inflammation [223]. In order to realize hydrogenothermal therapy in the NIR-II region, Jia et al. [107] first synthesized hollow carbon based on beta zeolite via template carbonization-corrosion process, followed by encapsulation of AB into the cavity (denoted as HC-AB NPs) (Fig. 8). The prominent absorbance in the NIR-II region indicated the capacities of HC-AB NPs as ideal NIR-II PAI CAs and PTAs (PCE = 25.45% at 1064 nm). Once entered into 4T1 cells, the HC-AB NPs underwent an acidity-response process to generate H_2_. Moreover, the production of H_2_ could be accelerated by the NIR-II PTT and in turn reduced the PTT-mediated inflammatory damage. Thanks to the synergistic manner, the HC-AB + Laser treatment displayed significant cell death and the tumors were destroyed without relapse in this group. These results revealed that the NIR-II hydrogenothermal treatment was much more effective than NIR-II PTT or gas therapy alone. Under the guidance of NIR-II PAI, the HC-AB NPs creates a bright future as a new NIR-II theranostic nanosystem.

## Hollow nanoplatforms for NIR-II PTT-based multi-modal therapies

Owing to the complex structure and changeable microenvironment of progressing tumors, even dual-modal therapies may sometimes cause unsatisfied curative outcomes [224, 225]. Therefore, extensive efforts have been devoted to fabricating more superior multi-modal therapies [226–228]. In this section, the synergistic NIR-II PTT/CDT/chemotherapy, PTT/chemo/gene therapy and PTT/PDT/CDT/ST/immunotherapy are presented.

### PTT/CDT/chemotherapy

**Fig. 9:**
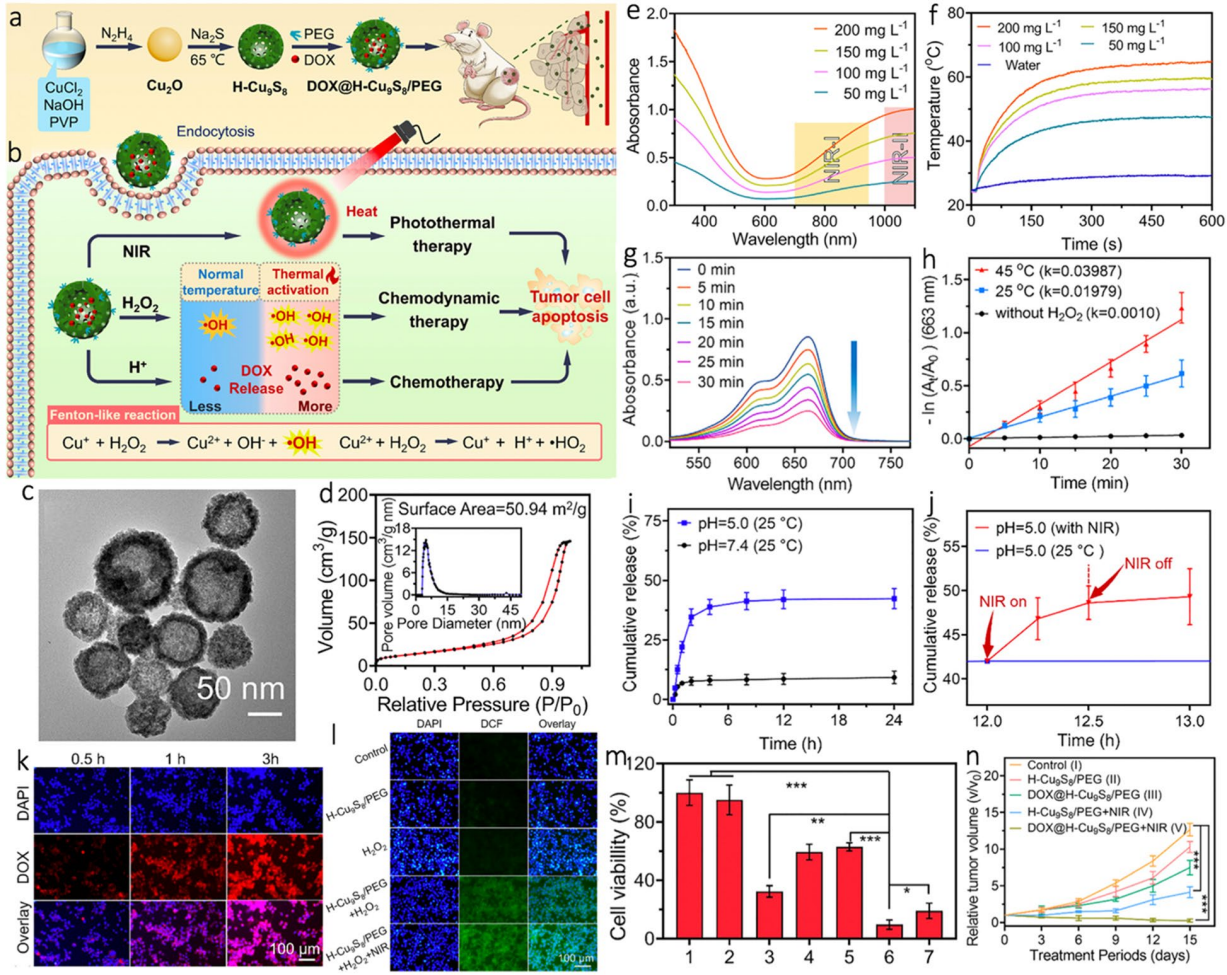
**a**, **b** A scheme illustrating the synthesis of DOX@H-Cu_9_S_8_/PEG with photothermally-augmented chemodynamic-chemo effect for the efficient killing of tumor cells. **c** TEM image of H-Cu_9_S_8_ NPs. **d** Isotherm analysis of H-Cu_9_S_8_ powder. **e** UV–vis–NIR spectra of H-Cu_9_S_8_/PEG dispersions (50–200 mg L^–1^). **f** Temperature elevation curves of H-Cu_9_S_8_/PEG (0–200 mg L^–1^) under the 1064 nm laser irradiation at 1.0 W cm^− 2^. **g** Time-dependent absorption spectra of MB mediated with H-Cu_9_S_8_/PEG and H_2_O_2_ upon laser irradiation (temperature balance: 45 °C). **h** The degradation rate of MB in Fenton-like reaction at 45 °C or ambient temperature (25 °C). **i**, **j** Cumulative release of DOX from DOX@H-Cu_9_S_8_/PEG with different treatments (*n* = 3). **k** Fluorescent images of CT26 cells incubated with DOX@H-Cu_9_S_8_/PEG for different durations (0.5, 1.0, and 3 h). **l** Fluorescent images of DAPI and DCFH-DA stained CT26 cells with different treatments. **m** Relative viabilities of CT26 cells after different treatments (*n* = 4). **n** Relative tumor volumes of mice after various treatments over 15 days (*n* = 4). Reproduced with permission from Ref. [108]. Copyright 2022, Elsevier

As we know, CuS NPs are widely used PTAs on account of the advantages including high PCE, good photostability, ease of synthesis, and low biological toxicity [229–231]. In the last few years, the Fenton-like catalytic activity of CuS gets noticed as well [232, 233]. As for hollow CuS NPs, the cavity also can be used for drug loading and delivery, providing multiple merits for cancer treatment if these functions are combined together. For example, Liu et al. [108] fabricated a multifunctional nanoplatform (denoted as DOX@H-Cu_9_S_8_/PEG) for NIR-II PTT/CDT/chemotherapy (Fig. 9a, b). As shown in Fig. 9c, the PEG coated hollow Cu_9_S_8_ NPs (H-Cu_9_S_8_/PEG NPs) were prepared through sulfurizing Cu_2_O NPs by Kirkendall effect, which exhibited an average diameter of ~ 100 nm and a shell thickness of ~ 10 nm. Besides, the Brunauer-Emmet-Teller (BET) analysis revealed the specific surface area and average pore size of H-Cu_9_S_8_ were 50.94 m^2^ g^− 1^ and 4.8 nm, respectively (Fig. 9d). These results confirmed the unique structure of H-Cu_9_S_8_ NPs that could be an ideal nanocarrier. The intensive NIR-II absorption endowed the H-Cu_9_S_8_ NPs with great potential as NIR-II PTAs (PCE = 40.9% at 1064 nm), in which a temperature elevation of 39.4 °C was found after irradiation for 10 min (1064 nm, 1.0 W cm^− 2^) when the concentration was 200 mg L^− 1^ (Fig. 9e, f). As revealed by the decrease of MB absorbance at 663 nm, the H-Cu_9_S_8_/PEG NPs were also able to conduct a Fenton-like reaction (Fig. 9 g). Notably, the Fenton-like process could be augmented by the photothermal effect, and the degradation rate of MB at 45 °C was increased by 2 times compared to that of control group (25 °C) (Fig. 9 h). Moreover, the inner hollow cavity gave the H-Cu_9_S_8_/PEG a high DOX loading capacity (21.1%), and the release of DOX was accelerated by acidic pH and hyperthermia (Fig. 9i, j). The vivid red fluorescence of DOX indicated the rapid cellular uptake and the strongest green fluorescence in the H-Cu_9_S_8_/PEG + H_2_O_2_ + NIR group was ascribed to the photothermal-boosted CDT (Fig. 9k, l). On account of the synergistic NIR-II PTT/CDT/chemotherapy, only 9.6% of the CT26 cells survived and the growth of CT26 tumor was efficiently suppressed as expected (Fig. 9 m, n).

Analogously, other photothermal Fenton agents with hollow structures also show great promise for PTT/CDT/chemotherapy. For example, Wang et al. [109] reported a novel hollow magnetite nanocluster (denoted as HMNC) for MRI-guided multimodal cancer treatment. In this design, pyrogenic decomposition of the ferric nitrate and urea complex first led to the formation of magnetite nanocrystals, then the nanocrystals aggregated to produce larger secondary solid magnetite nanoclusters (SMNCs), which lastly underwent the Ostwald ripening process to give HMNCs. The as-prepared HMNC proved to possess satisfied optical absorption for NIR-II PTT (PCE = 36.3% at 1064 nm) and the hollow structure provided a high DOX loading (~ 40%) for chemotherapy. Once faced the typical TME including low pH and overproduced H_2_O_2_, the HMNC would be degraded to trigger the DOX release and initiate the Fenton-like reaction to generate •OH for CDT. Moreover, these processes could be further promoted by the photothermal effect during PTT. Additionally, the inherent magnetic property enabled the HMNC to be good MRI CA with a calculated r_2_ of 62.97 mM^− 1^ s^− 1^. Both in vitro and in vivo studies evidenced the prominent antitumor efficiency and proper biosafety of HMNC-mediated triple-modal treatment under the guidance of T_2_-weighted MRI.

### PTT/chemo/gene therapy

**Fig. 10:**
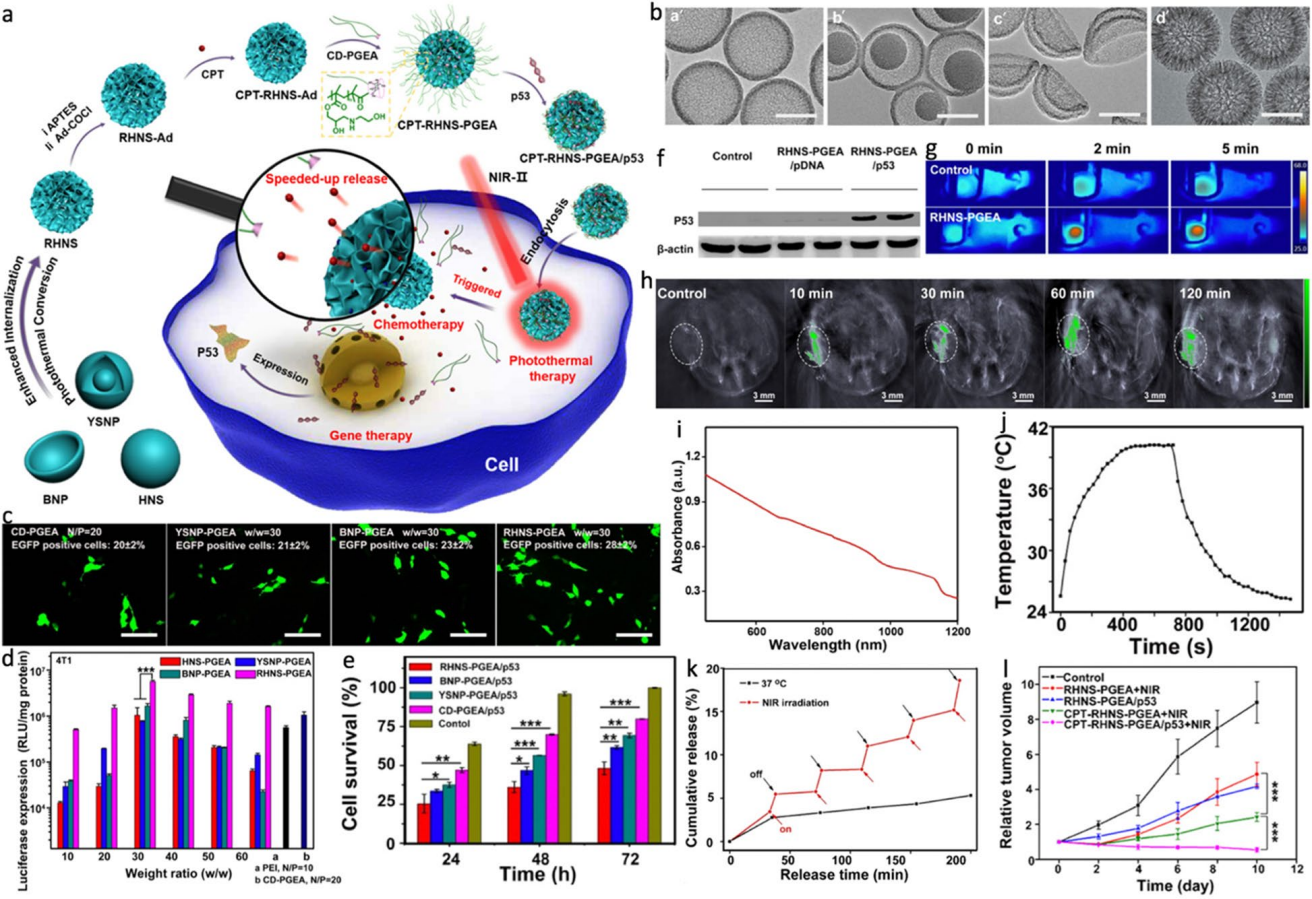
**a** Schematic illustration of the preparation and optimization of polycation–carbon nanohybrids with different morphologies (HNS, YSNP, BNP, and RHNS) for the NIR-II-responsive multimodal therapy. **b** TEM images of HNS (a′), YSNP (b′), BNP (c′), and RHNS (d′) (scale bar: 150 nm). **c** Representative fluorescence images of the EGFP expression of 4T1 cells treated with YSNP-PGEA, BNP-PGEA, and RHNS-PGEA/pDNA complexes at their optimal w/w ratio of 30 and CD-PGEA/pDNA at the optimal N/P ratio of 20. **d** In vitro luciferase expression of HNS-PGEA, YSNP-PGEA, BNP-PGEA, and RHNS-PGEA/pDNA complexes at various w/w ratios, in comparison with those of PEI (25 kDa) and CD-PGEA/pDNA complexes. **e** Cell proliferation of 4T1 cells treated with CD-PGEA, YSNP-PGEA, BNP-PGEA, and RHNS-PGEA/p53 complexes at 48 and 24/48/72 h, respectively. **f** Western blot analysis of the P53 expression in 4T1 cells after transfection with RHNS-PGEA/p53 complexes for 48 h. **g** Infrared thermal images of 4T1 tumor-bearing mice injected with PBS and RHNS-PGEA under laser irradiation. **h** PA images of 4T1 tumor-bearing mice before and at different time points after injection with RHNS-PGEA, where tumor regions are highlighted using white dashed circles. **i** Vis-NIR absorbance spectrum of the RHNS-PGEA solution (0.1 mg/mL). **j** Photothermal effect and cooling process of RHNS-PGEA in aqueous solution (Abs = 0.729 at 1064 nm) under 1064 nm laser irradiation (0.5 W cm^–2^). **k** Cumulative CPT release profile of CPT-RHNS-PGEA under laser irradiation at predetermined intervals. **l** 4T1 tumor growth curves of mice after various treatments. Reproduced with permission from Ref. [110]. Copyright 2022, American Chemical Society

Gene therapy that applies nucleic acids such as plasmid DNA (pDNA) and small interfering RNA (siRNA) to cure cancers is regarded to be a promising therapeutic method [234, 235]. However, several notable limitations including rapid enzymatic degradation and low intracellular uptake rate still limit the therapeutic efficacy [236, 237]. What’s worse, its strong negative charge faces an electrostatic barrier against internalization by cells [238, 239]. Combining PTT with gene therapy will be of significant promise, in which the hollow PTAs can act as gene delivery nanocarriers to effectively interact with negatively charged genes, resulting in significantly improved stabilities in serum and tumor accumulation [240, 241]. Antioncogene p53, encoding tumor-suppressor p53 protein, has been widely utilized for gene therapy of tumors [242]. By co-loading antioncogene p53 and chemotherapeutic drug onto hollow PTAs, triple-modal PTT/chemo/gene therapy can be obtained. For example, Zhao et al. [110] fabricated four kinds of carbon NPs-based organic/inorganic hybrid nanoplatform and investigate the impact of morphology on the therapeutic efficacy (Fig. 10a). By utilization tetraethylorthosilicate (TEOS) or tetrapropyl orthosilicate (TPOS) as silicon source, two kinds of resorcinol − formaldehyde (RF)-coated silica nanoparticles (SiO_2_@RF-1 and SiO_2_@RF-2) were obtained as the templates. As shown in Fig. 10b-d, hollow carbon nanospheres (HNS) and yolk − shell carbon nanoparticles (YSNP) showed similar hollow morphology while YSNP possessed a residual nanosized silica core because of its milder SiO_2_ corrosion step. Interestingly, the complete removal of silica component caused a sunken carbon nanoshell and resulted in bowl-like carbon nanoparticles (BNP). Unlike SiO_2_@RF-1, a great amount of silica primary particles existed in the outer surface of SiO_2_@RF-2, which would produce larger pores (rough surface) of the carbon nanoshell upon SiO_2_ removal via the corrosion step. Similar to HNS, rough hollow nanospheres (RHNS) were prepared from SiO_2_@RF-2, exhibiting consistent hollow nanoshell but a rather rough surface. After functionalization with a superior polycationic gene vector (denoted as CD-PGEA), the resultant RHNS-PGEA displayed the highest efficacy of gene transfection owing to the unique structure. With pRL-CMV plasmid (encoding Renilla luciferase) and pEGFP-N1 plasmid (encoding enhanced green fluorescent protein, EGFP) interacted, the RHNS-PGEA group exhibited more efficient luciferase and EGFP expression than the other three groups. As expected, the corresponding RHNS-PGEA/p53 complex displayed superior cell apoptosis due to the effective gene therapy (Fig. 10e, f). Thanks to the considerable NIR-II absorption, the RHNS-PGEA showed excellent photothermal effect with a calculated PCE of 59.2% upon a 1064 nm laser irradiation, which was higher than that of HNS-PGEA (42.8%), YSNP-PGEA (45.4%) and BNP-PGEA (38.4%) (Fig. 10 g, h). Such outstanding performance not only enabled HNS-PGEA to be ideal PTT/PAI nanoagents but also a smart switch to control the release of encapsulated 10-hydroxy camptothecin (CPT) (Fig. 10i-k). Taken together, remarkable treatment outcomes could be realized by PAI-guided triple-modal NIR-II PTT/gene/chemotherapy (Fig. 10 L). This study proposes a new strategy for rationally designing nanohybrids with advantageous morphology as multifunctional cancer nanotheranostics.

### PTT/PDT/CDT/ST/immunotherapy

Immunotherapy has been fundamentally changing the landscape of clinical cancer treatment, which profits handsomely from research progress in cancer biology as well as anticancer immunity, especially the discovery of several dominant immunosuppressive pathways [243, 244]. These groundbreaking advances were recognized by the 2018 Nobel Prize in Physiology or Medicine that was awarded to James Allison and Tasuku Honjo for “the discovery of cancer therapy by inhibition of negative immune regulation” [245, 246]. In particular, the Nobel prize was awarded for the identification of immune checkpoints including cytotoxic T lymphocyte-associated antigen 4 (CTLA4) and programmed cell death-1/programmed death-ligand 1 (PD-1/PD-L1), leading the research boom for anticancer therapy by targeting these checkpoints [247]. In addition, nanomedicines prove to trigger the induction of immunogenic cell death (ICD), which is a specific mode of cell death with tumor antigens and danger-associated molecular patterns released to boost anticancer immunity [248, 249]. Nowadays, various kinds of therapeutic modalities such as chemotherapy, PTT, PDT and radiotherapy have been reported to induce ICD. Thus, the integration of nanomedicine with immunotherapy opens a new research tendency in curing cancers [250–252].

**Fig. 11:**
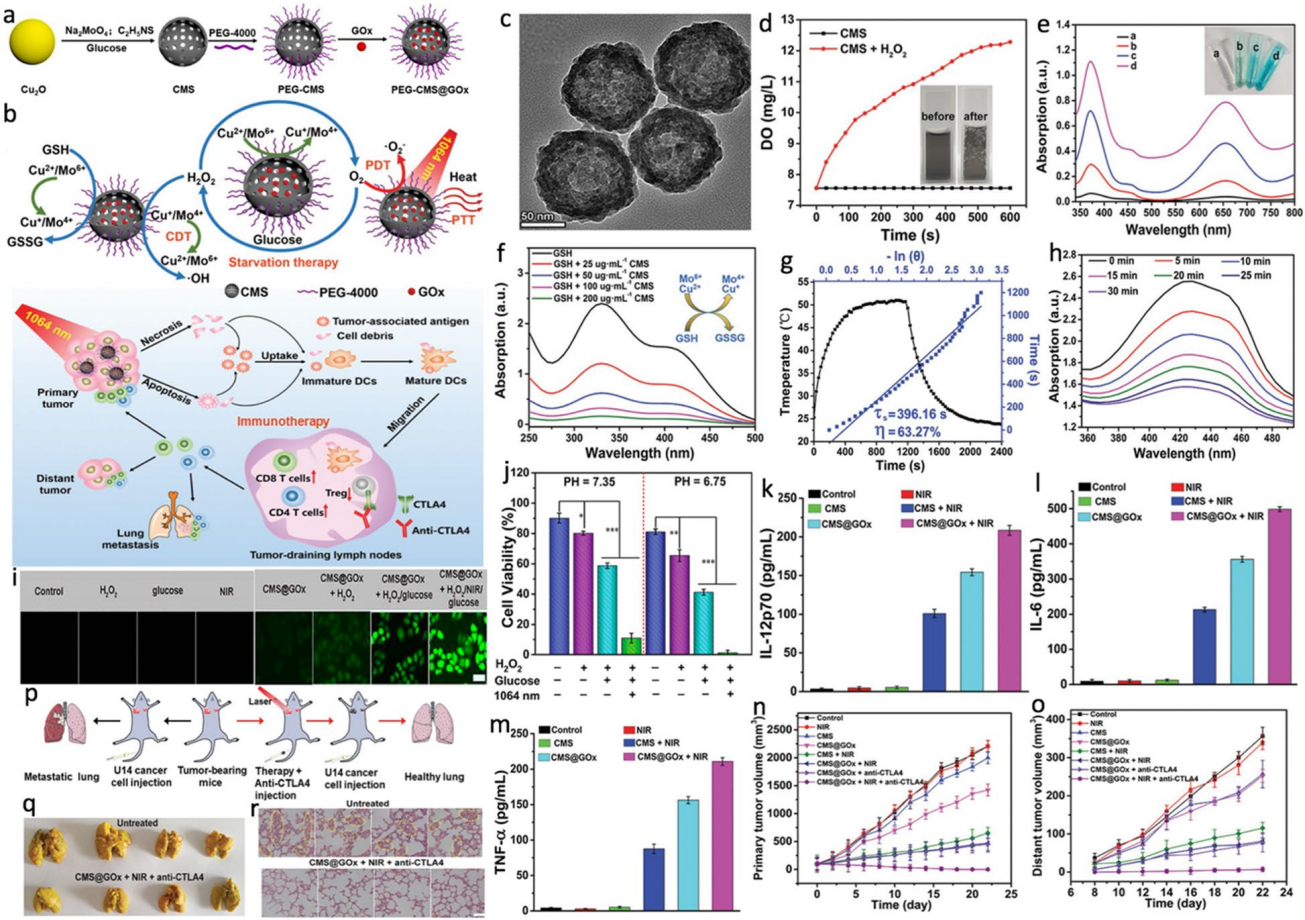
**a** The schematic illustration of synthetic process of PEGylated CMS@GOx. **b** Schematic illustration of fabrication and mechanism of PEGylated CMS@GOx for PTT/PDT/CDT/ST and the mechanism of antitumor immune responses induced by PEGylated CMS@GOx-based phototherapy in combination with checkpoint blockade therapy. **c** TEM image of CMS. **d** O_2_ generation curve of CMS aqueous solution (200 µg mL^− 1^, pH = 6.75) without and with H_2_O_2_ addition (100 × 10^− 6^ M). Illustrations are O_2_ generation photographs of CMS with H_2_O_2_ addition before and after 1 h. **e** H_2_O_2_ generation in CMS@GOx solution arising from the addition of different concentrations of glucose (insert a-d: 0 × 10^− 3^ M, 2 × 10^− 3^ M, 4 × 10^− 3^ M and 8 × 10^− 3^ M). **f** GSH depletion (89 × 10^− 6^ M) under the reduction of different concentrations of CMS. **g** Heating and cooling curves of CMS aqueous solution (200 µg mL^− 1^, 1 mL) under 1064 nm (0.48 W cm^− 2^) laser irradiation, linear time data obtained from the cooling period. **h** Depletion of DPBF over CMS due to •O_2_^−^ generation (65 µg mL^− 1^ of CMS, 0.48 W cm^− 2^ of 1064 nm laser). **i** The inverted fluorescence microscopy images of ROS production after different treatments (scale bars: 20 μm). **j** Assessment of synergistic comprehensive treatment effects of PEGylated CMS@GOx by the MTT assay. *p < 0.05, **p < 0.01, and ***p < 0.001 (two-tailed t-test). **k-m** The secretion levels of IL-12p70, IL-6, and TNF-α by ELISA assay in DC suspensions after different treatments. **n**, **o** Growth curves of primary tumor volume and distant tumor volume in Balb/c mice with different treatments. **p** Scheme of PEGylated CMS@GOx-based synergistic comprehensive treatment combined with anti-CTLA4 checkpoint blockade treatment for lung metastasis inhibition in Balb/c mice. **q** Representative images of picric-acid-stained lung tissues from different treatment groups, with metastatic nodules indicated by red circles. **r** H&E staining of lung tissues with different treatments. The yellow circles in the figure show the lung metastatic lesions (scale bar: 20 μm). Reproduced with permission from Ref. [111]. Copyright 2019, Wiley-VCH.

For example, Chang et al. [111] fabricated a versatile cascade nanoreactor (denoted as PEG-CMS@GOx) consisted of hollow mesoporous copper molybdenum sulfide (Cu_2_MoS_4_, denoted as CMS) and GOx with PEG modified for multi-modal cancer therapy (Fig. 11a, b). As shown in Fig. 11c, the as-synthesized CMS had obvious hollow mesoporous structures with a pore size ranging from 3.15 to 9.84 nm, which could be an ideal nanocarrier for delivering GOx. Moreover, the CMS displayed typical catalase-mimicking performance to produce O_2_ from catalytic decomposition of H_2_O_2_ to support the GOx-mediated glycolysis for ST (Fig. 11d). Owing to the existence of multivalent elements (Cu^1+^/^2+^, Mo^4+^/^6+^), the CMS exhibited good Fenton-like activity and GSH depleting capacity, resulting in accelerated •OH generation for CDT (Fig. 11e, f). Furthermore, upon exposure to a 1064 nm laser, the CMS showed excellent photothermal and photodynamic effects, producing hyperthermia (PCE = 63.3% at 1064 nm) and cytotoxic •O_2_^−^ for NIR-II PTT/PDT (Fig. 11 g, h). More importantly, the regenerated H_2_O_2_ by consuming glucose and heat from PTT could further promote the CDT (Fig. 11i). Nearly 100% of the HeLa cells were killed after PEG-CMS@GOx + NIR (1064 nm, 0.48 W cm^− 2^, 5 min) treatment due to the synergistic PTT/PDT/CDT/ST (Fig. 11j). In addition, such remarkable treatment efficacy was able to significantly induce in vitro dendritic cells; IL-12p70 (DCs) maturation and trigger strong immune responses, leading to the improved secretion of diverse cytokines including interleukin 12 (IL-12p70), interleukin 6 (IL-6) and tumor necrosis factor α (TNF-α) (Fig. 11k-m). To further boost the antitumor capacity, checkpoint inhibitor anti-CTLA4 was integrated and the in vivo therapeutic effects were investigated on the U14 primary/distant tumors-bearing mice. As shown in Fig. 11n-o, the PEGylated CMS@GOx + NIR + anti-CTLA4 group displayed the most superior curative effects, which not only ablated primary tumor but also prominently suppressed the distant tumor growth. Further study based on the aggressive lung metastasis model also indicated that the combination of synergistic PTT/PDT/CDT/ST with CTLA4 blockade therapy could inhibit lung metastasis (Fig. 11p-r). This work reports an innovative paradigm for comprehensive cancer treatment, showing extraordinary value for future clinical translation.

## Conclusion

Over the past decade, PTT especially NIR-II PTT has attracted ever-increasing attention due to its unique advantages. To compensate single-modal PTT for the deficient curative outcome, different treatment methods collecting various prominent merits are combined with PTT to achieve superior anticancer effects. Hollow nanoplatforms appear to be promising for practical applications with remarkable merits being noticed in their special material properties and structures, the fruitful utilization of which can realize multifunctional all-in-one theranostics. In this review, a detailed summary of the latest hollow PTAs for single-modal NIR-II PTT, dual-modal NIR-II PTT/PDT, PTT/chemotherapy, PTT/catalytic therapy and PTT/gas therapy as well as multi-modal NIR-II PTT/CDT/chemotherapy, PTT/chemo/gene therapy and PTT/PDT/CDT/ST/immunotherapy is presented. In addition to intrinsic PAI performance of PTAs, these hollow nanoplatforms also can be used for MRI, FLI and CT imaging. For better comparison, the materials, synthetic methods/mechanisms, PCE values, cancer cell types/tumor models as well as biomedical applications of each hollow PTAs are described in Table 1. Though exciting progresses have been made with the endeavor of researchers in nanoscience, chemistry, physics and medicine, several key issues still remain to be solved before clinical translation of these avant-garde nanotheranostics from the bench to bedside.

**Table 1:** Summary of various NIR-II hollow nanoplatforms for photothermal-based cancer theranostics (PTT refers to NIR-II PTT)

Material	Templates and mechanisms	NIR-II PTAs and PCE	Tumor model	Biomedical applications	Reference
HPP	SiO_2_	HPP; 45.1%	4T1 tumor	PTT	[97]
Ag_2_S Ve	self-assembly	Ag_2_S QDs; —	4T1 tumor	NIR-II FLI and PTT	[98]
AuHNRs-DTPP	Te nanorods	AuHNRs; —	H22 tumor	FLI and PTT/PDT	[99]
AAM-Ce6	galvanic replacement reaction	AAM-Ce6; 52.5%	HeLa tumor	PAI/FLI/MRI and PTT/PDT	[100]
TAT-Pd@Au/Ce6/PAH/H-MnO_2_	mSiO_2_	TAT-Pd@Au; 56.9%	MCF-7 tumor	T_1_-weighted MRI and PTT/PDT	[101]
AuHNRs-DOX	Se-doped Te nanorod	AuHNRs; 33%	SCC-7 tumor	PAI/CT imaging and PTT/chemotherapy	[102]
NiP PHNPs-DOX	HCl solution etching	NiP PHNPs; 56.8%	U14 tumor	T_1_-weighted MRI/PAI and PTT/chemotherapy	[103]
DSF@PEG-HCuS	nanoscale Kirkendall effect	DSF@PEG-HCuS; 23.8%	4T1 tumor	PAI and PTT/chemotherapy	[104]
PEG-Cu_2_Se HNCs	anion exchange method	Cu_2_Se HNCs; 50.89%	4T1 tumor	PTT/CDT	[105]
HSC-2	nano-Beta zeolite	HSC-2; 41.41%	4T1 tumor	NIR-II PAI/FLI and PTT/catalytic therapy	[106]
HC-AB	beta zeolite	HC-AB; 25.45%	4T1 tumor	NIR-II PAI and PTT/gas therapy	[107]
DOX@H-Cu_9_S_8_/PEG	nanoscale Kirkendall effect	H-Cu_9_S_8_; 40.9%	CT26 tumor	NIR-II PAI and PTT/CDT/chemotherapy	[108]
HMNC	Ostwald ripening process	HMNC; 36.3%	HeLa tumor	T_2_-weighted MRI and PTT/CDT/chemotherapy	[109]
CPT-RHNS-PGEA/p53	SiO_2_-RF-1	RHNS-PGEA; 59.2%	4T1 tumor	PAI and PTT/chemo/gene therapy	[110]
PEG-CMS@GOx	nanoscale Kirkendall effect	CMS; 63.3%	U14 tumor	PTT/PDT/CDT/ST/ immunotherapy	[111]

When comes to clinical application, biosafety and biomedical effect are of primary significance. The PTAs involved in NIR-II PTT should be biocompatible and possess low toxicity without NIR light radiation. In this regard, diverse polymers and proteins such as PEG and BSA are used for surface modification to improve their physiological stability and biocompatibility, but the lack of cancer specific units in the above-mentioned researches leads to unsatisfactory tumor accumulation. Therefore, further studies are required to focus on constructing various active targeting nanoplatforms to improve the diagnostic and therapeutic efficacy. Meanwhile, more attention should be pained in developing ultrasmall and/or biodegradable and clearable PTAs and/or hollow matrices that can be extracted from the body after treatment. So far, the majority of animal models are mice, the investigations based on large animal models like primates are urgently needed. PCE is the key factor for efficient PTT, previous reports have proved that the adjustment of size and shape as well as the doping of heterogeneous ions result in higher PCE values [37, 45]. Notably, the pore structures and cavity volume of hollow nanoplatforms are beneficial for incorporating different functions, more efforts should be made to explore their internal connections to achieve synergistic manners rather than just simply combine them. To meet clinical demand, the mass production of NIR-II hollow nanoplatforms by a facile and mild synthetic way is of significant importance. The self-templating strategy tends to be more attractive due to the advantages including ease of preparation and reduced formation of chemical waste in contrast to the sacrificial-template-based strategy. With the continuing efforts of scientists from different fields, we believe that these NIR-II hollow nanotheranostics will reach their full potential for clinical translation in the near future.

## Data Availability

Data sharing is not applicable to this article as no datasets were generated or analysed during the current study.
